# *Staphylococcus aureus* inhibits the NLRP3 inflammasome in macrophages to varying degrees during early and late stages of infection

**DOI:** 10.1128/iai.00200-26

**Published:** 2026-06-10

**Authors:** Saumya Bhagat, Khushpreet Kaur, Jenna R. Petronglo, Luke O'Connor, Chun Wang, Yongjia Li, Gaurav Swarnkar, Kunjan Khanna, James E. Cassat, Deborah J. Veis, Gabriel Mbalaviele

**Affiliations:** 1 Division of Bone & Mineral Diseases, Musculoskeletal Research Center, Washington University School of Medicine 12275, St. Louis, Missouri, USA; 2 Department of Pathology, Microbiology, and Immunology, Vanderbilt University Medical Center 204907, Nashville, Tennessee, USA; 3 Department of Orthopedic Surgery, Washington University School of Medicine 12275, St. Louis, Missouri, USA; 4 Department of Biomedical Engineering, Vanderbilt University 575178, Nashville, Tennessee, USA; 5 Vanderbilt Institute for Infection, Immunology, and Inflammation, Nashville, Tennessee, USA; 6 Department of Pathology and Immunology, Washington University School of Medicine 12275, St. Louis, Missouri, USA; St Jude Children's Research Hospital, Memphis, Tennessee, USA

**Keywords:** *Staphylococcus aureus*, bone marrow-derived macrophages, NLRP3 inflammasome, pyroptosis, IL-1β, gasdermin D, caspase-1, osteomyelitis, host-pathogen interaction, infection

## Abstract

*Staphylococcus aureus* (*S. aureus*) is a highly virulent pathogen responsible for chronic infections such as osteomyelitis. Although its interaction with the host immune system has been widely studied, the specific role of inflammasomes in regulating the infection within macrophages remains unclear. We investigated this question using bone marrow-derived macrophages (BMDMs) infected with *S. aureus* for up to 96 hours (h). We observed a significant reduction in intracellular bacterial load beginning at 18 h post-infection (hpi), which continued through 96 hpi; this response was partially attenuated in BMDMs lacking NLRP3. Mechanistically, activation of the NLRP3 inflammasome, including ASC speck formation, IL-1β secretion, and pyroptosis, was modest and detectable only after 18 hpi. Consistent with these findings, infected BMDMs failed to respond robustly to NLRP3 inflammasome activators, lipopolysaccharide (LPS) and nigericin, up to 18 hpi, with only partial restoration of responsiveness at later time points. We also found that NLRP3 deficiency did not significantly affect bacterial burden control or bone homeostasis in experimental *S. aureus* osteomyelitis. Thus, *S. aureus* is inadequately controlled by the NLRP3 inflammasome in macrophages and within the bone microenvironment.

**IMPORTANCE:**

*Staphylococcus aureus* causes difficult-to-treat, long-lasting infections such as osteomyelitis. While many aspects of how the immune system responds to this pathogen are known, it is still unclear how the inflammasomes contribute to controlling the infection inside macrophages. In this study, we found that macrophages gradually reduced the number of bacteria they contained over time, but this ability was only partly dependent on one major inflammasome component, NLRP3. Importantly, the inflammasome response to *S. aureus* was weak and delayed, becoming noticeable only well after infection had begun. Even when stimulated with agents that normally trigger a strong inflammasome response, infected macrophages responded poorly. Consistent with these findings, the absence of NLRP3 did not significantly worsen bacterial control or bone damage in a mouse model of osteomyelitis. Together, these results suggest that *S. aureus* largely evades inflammasome-based defenses, limiting their effectiveness in controlling infection in immune cells and bone.

## INTRODUCTION

*Staphylococcus aureus (S. aureus*) is a highly versatile gram-positive pathogen responsible for a wide range of infections, including osteomyelitis (1). Commonly considered an extracellular pathogen, *S. aureus* also survives within host cells (2). Its ability to infect, survive, and propagate in the bone microenvironment within various cell types, including macrophages, osteoclasts, neutrophils, and mesenchymal cells, is central to the pathogenicity of chronic osteomyelitis (3). The ability of *S. aureus* to thrive as a pathogen can be largely attributed to its extensive array of virulence factors, which allow the bacterium to efficiently extract nutrients from its host while evading both innate and adaptive immune defenses (4, 5).

Macrophages are pivotal in the immune responses against pathogens, serving as the first line of defense through phagocytosis and the initiation of inflammatory responses. They are equipped with a variety of pattern recognition receptors, such as Toll-like receptors and NOD-like receptors (NLRs), which are essential for detecting pathogen-associated molecular patterns (PAMPs) (6). They are also highly efficient at phagocytosing *S. aureus,* which, upon internalization, is enclosed in phagosomes that subsequently fuse with lysosomes to form phagolysosomes (7). The acidic environment within the phagolysosome, along with reactive oxygen species (ROS) and reactive nitrogen species, contributes to the destruction of the bacteria (8, 9). Additionally, macrophages produce a wide range of cytokines and chemokines in response to *S. aureus* infection, such as TNF-α, IL-1β, and IL-6, which help recruit additional immune cells to the site of infection and amplify the inflammatory responses (10, 11). However, *S. aureus* has evolved several strategies to evade killing within macrophages. For example, it produces catalase, which neutralizes ROS, and proteins that inhibit phagosome-lysosome fusion, allowing the bacterium to survive and replicate intracellularly (12). Given the immune functions of macrophages, it stands to reason that evasion of macrophage-dependent killing is required to successfully establish and maintain a productive infection.

The inflammasomes assembled by NLR family pyrin domain-containing 3 (NLRP3) and absent in melanoma 2 (AIM2) play critical roles in host defense against *S. aureus* infection. NLRP3 detects bacterial toxins and lipoproteins, which cause membrane damage, lysosomal rupture, and ROS production, thereby triggering inflammasome activation to control the infection (13–15). The AIM2 inflammasome is particularly important in fighting intracellular bacterial pathogens such as *S. aureus*, *L. monocytogenes*, *F. tularensis,* and *M. tuberculosis* upon the detection of DNA in the cytoplasm, which originates from lysed bacteria (16, 17). Although activated through distinct mechanisms, both NLRP3 and AIM2 assemble macromolecular protein complexes that include apoptosis-associated speck-like protein containing a CARD (ASC) and caspase-1 (18–20). Caspase-1 not only processes pro-IL-1β and pro-IL-18 into their active forms but also cleaves gasdermin D (GSDMD), generating amino-terminal fragments that form plasma membrane pores through which these cytokines are secreted (21–23).

Despite extensive research, several key elements regarding *S. aureus* and inflammasome activation remain unclear, including the precise mechanisms by which *S. aureus* modulates inflammasome activity during acute and chronic infections, the contribution of alternative inflammasome pathways beyond NLRP3 in the detection and response against *S. aureus*, and the balance between protective immune responses and pathological inflammation in diseases such as osteomyelitis and sepsis. Filling this knowledge gap is essential for designing targeted therapies that enhance immunity while minimizing tissue damage.

In this study, we studied the interactions between *S. aureus* and BMDMs. We identified NLRP3 but not AIM2 as the primary sensor of this bacterium in BMDMs. We found that *S. aureus* initially suppresses NLRP3 inflammasome activation, and as infection progresses, BMDMs only partially overcome this inhibition. Thus, *S. aureus* is insufficiently controlled by NLRP3 inflammasome activity in macrophages and within the bone microenvironment.

## RESULTS

### NLRP3 inflammasome contributes to the time-dependent killing of *S. aureus* by BMDMs

BMDMs exhibit robust antimicrobial activity and efficiently mediate intracellular clearance of *S. aureus*. Fetal bovine serum (FBS) supports the *in vitro* survival, growth, and immune responses of phagocytes, including macrophages (24, 25). To evaluate the impact of serum supplementation on the ability of BMDMs to clear *S. aureus* infection, WT BMDMs were cultured in media containing 0%, 1%, or 10% FBS and subsequently infected with *S. aureus* USA300 clinical isolate Ti3 strain (hereafter referred to as *S. aureus*) at a multiplicity of infection (MOI) of 100. The CFU assay showed a decrease in bacterial load at 18 hpi compared to 1.5 hpi across all conditions, with significantly greater clearance observed with 10% FBS supplementation than with 0% FBS, underscoring the impact of serum on bacterial elimination (Fig. S1a).

The inflammasomes are induced by *S. aureus* and are responsible for the maturation and secretion of IL-1β, and they can also induce pyroptosis (13, 26). Therefore, we analyzed the secretion of IL-1β and lactate dehydrogenase (LDH; a readout of cell death). LDH release was significantly higher at 18 hpi compared to 1.5 hpi across all conditions, with the maximal response observed in cultures containing 10% FBS (Fig. S1b). By contrast, IL-1β levels were only detected in cells exposed to 1% or 10% FBS, with the highest response observed in the 10% FBS group (Fig. S1c). BMDMs treated with LPS and nigericin served as positive controls (Fig. S1d).

**Fig 1:**
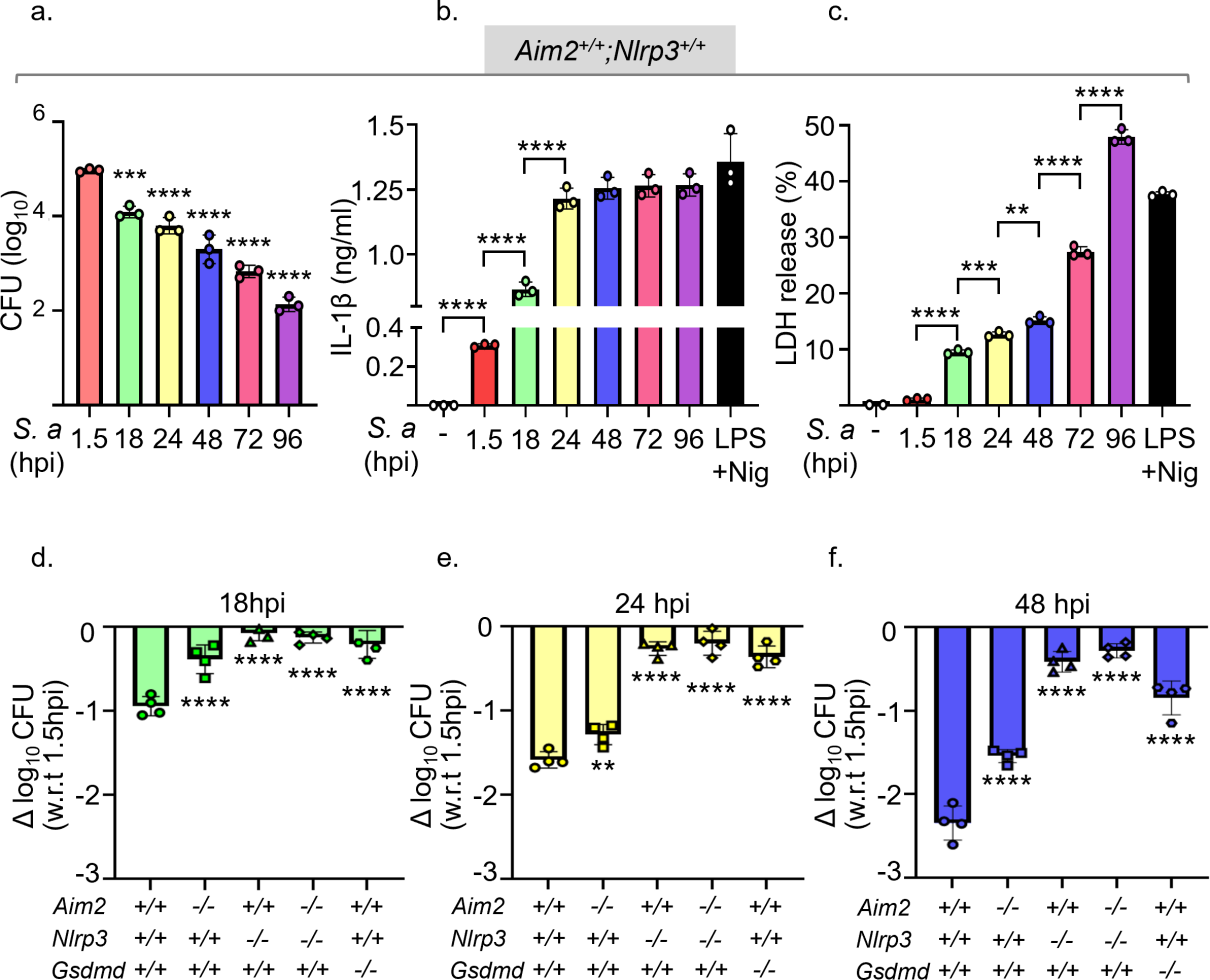
The killing of *S. aureus* by BMDMs is time- and NLRP3 inflammasome-dependent. *Aim2^+/+^;Nlrp3^+/+^*, *Aim2^+/+^;Nlrp3^-/-^, Aim2^-/-^;Nlrp3^+/+^, Aim2^-/-^;Nlrp3^-/-^,* and *Gsdmd^-/-^* BMDMs were infected with *S. aureus* for up to 96 h. Cells treated sequentially with LPS for 3 h and nigericin for 45 min served as positive controls. Time point analysis of WT BMDMs was assessed by (**a**) CFU, (**b**) IL-1β secretion, and (**c**) LDH release. (**d–f**). CFU assessment was also done for knockout genotypes and compared to WT, and data were normalized to 1.5 hpi internalization time point. Data are means ± SD from three biological replicates and represent combined results from three independent experiments. **P < 0.01; ***P < 0.001; ****P < 0.0001 by one-way ANOVA with Bonferroni’s *post hoc* test.

Given that BMDMs exhibit optimal control of *S. aureus* infection, LDH release, and IL-1β secretion in the presence of 10% FBS, we leveraged this condition to assess their ability to clear this bacterium and secrete IL-1β over time, up to 96 hpi. The number of CFUs declined in a time-dependent manner (Fig. 1a), a pattern that inversely correlated with the secretion of IL-1β, which peaked at 24 hpi (Fig. 1b), and LDH release (Fig. 1c). To determine whether these observations were Ti3 strain-specific, we included additional USA300 derivatives, namely the LAC and JE2 strains, which have been linked to the NLRP3 inflammasome (27, 28). JE2, lacking carotenoid desaturase N (Δ*crtN* JE2) and α-hemolysin-deficient LAC (Δ*hla* LAC), alongside heat-killed Ti3 (Ti3 HK), was also tested as a Ti3 mutant was unavailable. At comparable MOIs and despite similar internalization at 1.5 hpi, WT Ti3, HK Ti3, and WT LAC induced similar levels of IL-1β secretion, albeit to a lesser extent than WT JE2 (Fig. S2). Notably, Δ*hla* LAC and Δ*crtN* JE2 elicited significantly higher IL-1β secretion compared to the respective WT strains. Overall, these data highlight strain-specific differences in BMDM responses to *S. aureus* and support a role for hla and crtN in limiting inflammasome activation. To further understand the underlying inhibitory mechanisms, we focused on the WT Ti3 strain.

To investigate the role that inflammasomes play in the fate of *S. aureus* in our experimental models, we enumerated CFUs in BMDMs expressing or lacking AIM2 or NLRP3, which are both well known for sensing this bacterium (29–31). The ability of *Aim2^+/+^;Nlrp3^+/+^* BMDMs to clear *S. aureus* infection increased in a time-dependent manner (Fig. 1d through f), consistent with the results shown in Fig. 1a. Although the killing of *S. aureus* by *Aim2^-/-^;Nlrp3^+/+^* BMDMs was slightly impaired at 18 hpi, this response was almost fully restored in these cells at later time points (Fig. 1d through f; Fig. S3a). In contrast, *Aim2^+/+^;Nlrp3^-/-^* BMDMs and *Aim2^-/-^;Nlrp3^-/-^* counterparts showed reduced clearance of this bacterium (Fig. 1d through f; Fig. S3a). Comparable results were obtained when the fate of *S. aureus* in WT and mutant BMDMs was measured by flow cytometry (Fig. S3b and c). GSDMD is processed alongside pro-IL-1β upon AIM2 or NLRP3 inflammasome activation (22). Therefore, we also determined the impact of GSDMD deficiency on the fate of *S. aureus* in BMDMs. The number of CFUs did not change significantly over time in *Gsdmd^-/-^* BMDMs (Fig. 1d through f; Fig. S3a). These results suggest that the NLRP3 inflammasome-GSDMD axis contributes to *S. aureus* killing by BMDMs.

**Fig 2:**
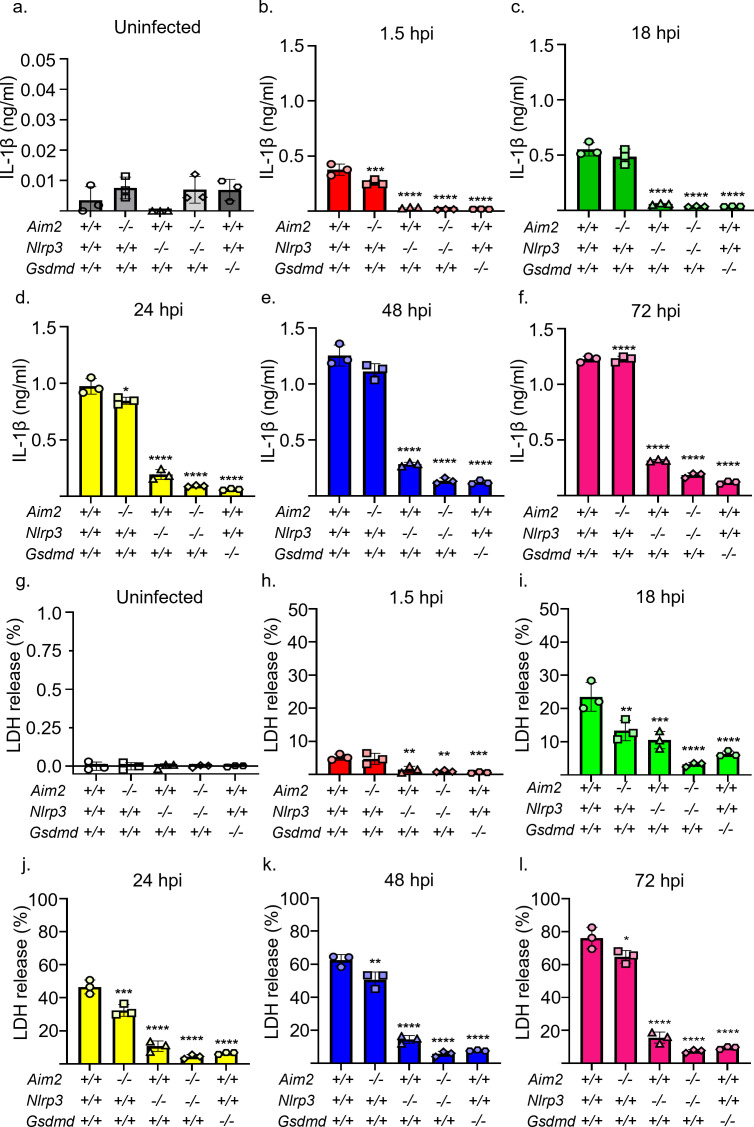
*S. aureus*-induced IL-1β secretion and LDH release are time- and NLRP3 inflammasome-dependent. *Aim2^+/+^;Nlrp3^+/+^*, *Aim2^+/+^;Nlrp3^-/-^, Aim2^-/-^;Nlrp3^+/+^, Aim2^-/-^;Nlrp3^-/-^,* and *Gsdmd^-/-^* BMDMs were left uninfected or infected with *S. aureus* for up to 96 h. Uninfected cells sequentially treated with LPS for 3 h and nigericin for 45 min were used as positive controls. The supernatants were analyzed for IL-1β secretion and LDH release. (**a–f**) IL-1β secretion. (**g–l**) LDH release. Data are means ± SD from three biological replicates and are representative of at least three independent experiments. **P < 0.01; ***P < 0.001; ****P < 0.0001 by one-way ANOVA with Bonferroni’s *post hoc* test.

We also monitored the kinetics of IL-1β secretion and LDH release during the progression of BMDM infection by *S. aureus*. The levels of secreted IL-1β by WT BMDMs were low at 1.5 hpi, but increased over time, reaching a maximum at 48 hpi (Fig. 2a through f; Fig. S4a). Consistent with CFU results, while the levels of IL-1β were only marginally altered in *Aim2^-/-^;Nlrp3^+/+^* BMDMs compared to *Aim2^+/+^;Nlrp3^+/+^* cells, they were markedly reduced at all time points in Aim2^+/+^;*Nlrp3^-/-^*, *Aim2^-/-^;Nlrp3^-/-^*, or *Gsdmd^-/-^* BMDMs (Fig. 2a through f). Likewise, the release of LDH by *Aim2^+/+^;Nlrp3^+/+^* and *Aim2^-/-^;Nlrp3^+/+^* BMDMs, which was minimal at 1.5 hpi, increased progressively thereafter, and then plateaued at 48 hpi (Fig. 2g through l). These outcomes were significantly inhibited in *Aim2^+/+^;Nlrp3^-/-^*, *Aim2^-/-^;Nlrp3^-/-^*, or *Gsdmd^-/-^* BMDMs (Fig. 2g through l). IL-18 levels, unlike those of IL-1β, increased only modestly during *S. aureus* infection (Fig. S4b and c). Collectively, these findings indicate that NLRP3 is more active than AIM2 in sensing *S. aureus*, with inflammasome responses increasing as infection progresses.

### *S. aureus* inhibits the NLRP3 inflammasome in macrophages to varying degrees during early and late stages of infection

**Fig 3:**
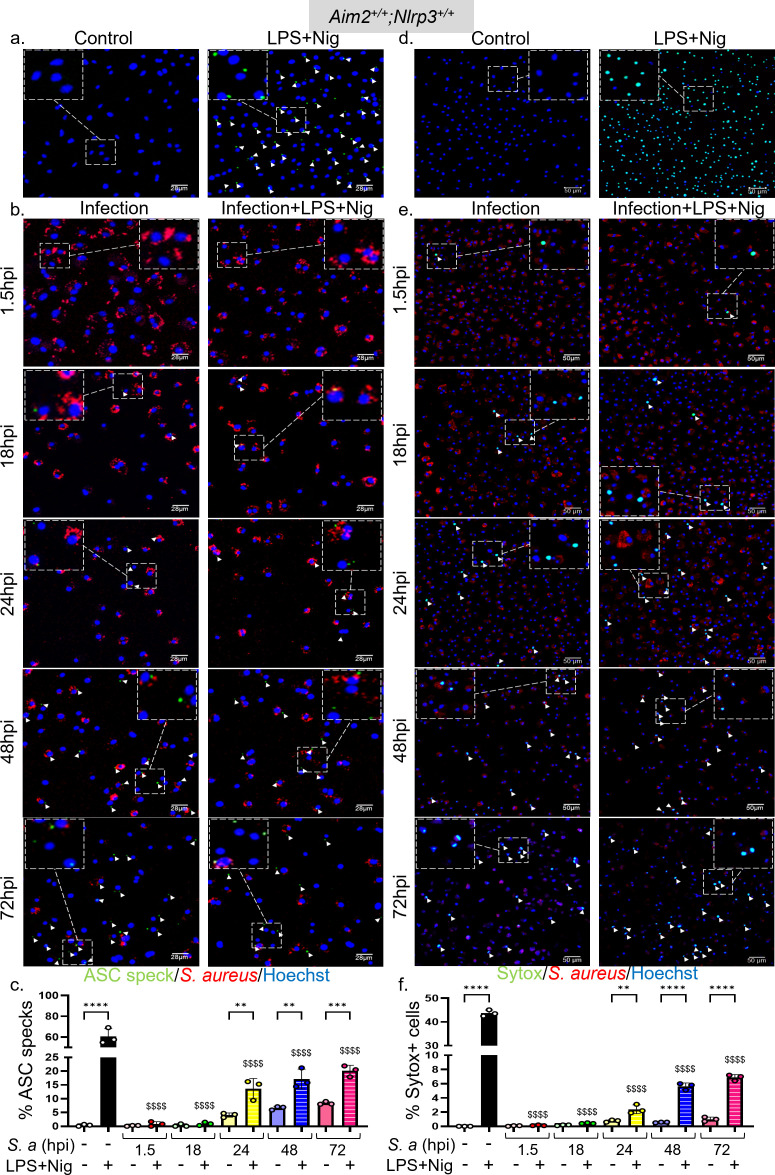
*S. aureus* suppresses ASC speck formation and Sytox Green uptake during the early phases of infection of BMDMs, but to a lesser extent during the late phases. BMDMs from *ASC-citrine* mice (to assess ASC speck formation) and WT mice (to measure Sytox Green uptake) were infected with *S. aureus* for up to 72 h. Uninfected left untreated or treated sequentially with LPS for 3 h and nigericin for 45 min of nigericin were used as controls. The cells were incubated with Hoechst 33342 (1 µg/mL) for 15 min after LPS treatment. (**a, b**) ASC specks were visualized under a fluorescence microscope and (**c**) quantified using ImageJ. (**d, e**) Sytox Green was added to the cultures 10 min prior to imaging by confocal microscope, and (**f**) the uptake was quantified using ImageJ. Data are means ± SD from three biological replicates and are representative of at least three independent experiments. **P < 0.01; ***P < 0.001; ****P < 0.0001 by one-way ANOVA with Bonferroni’s *post hoc* test. Comparison of LPS + Nig-positive control with infection samples treated with LPS + Nig shown as $$$$ *P* < 0.0001 by one-way ANOVA with Bonferroni’s post hoc test.

As mentioned above, upon activation, NLRP3 undergoes oligomerization and recruits ASC, which then forms polymers. These macrostructures, known as ASC specks, can be visualized as foci under a fluorescence microscope (18). Uninfected BMDMs from Asc-citrine mice robustly formed ASC specks following treatment with LPS and nigericin, whereas untreated cells did not (Fig. 3a; Fig. S5). By contrast, ASC specks in *S. aureus*-infected BMDMs were detected only after 18 hpi and were present in approximately 5% of cells (Fig. 3b and c). Given the delay in ASC speck formation, we hypothesized that the NLRP3 inflammasome was suppressed during the initial stages of infection. To evaluate this hypothesis, we examined the activation of this inflammasome in response to LPS and nigericin in both uninfected and infected cells. While approximately 60% of uninfected BMDMs treated with LPS and nigericin formed ASC specks, this response was blunted in infected cells up to 18 hpi and was only partially restored at 24, 48, and 72 hpi (Fig. 3a through c). Similar trends of inflammasome activation were observed when we monitored the activated state of caspase-1 (Fig. S6a and b).

**Fig 4:**
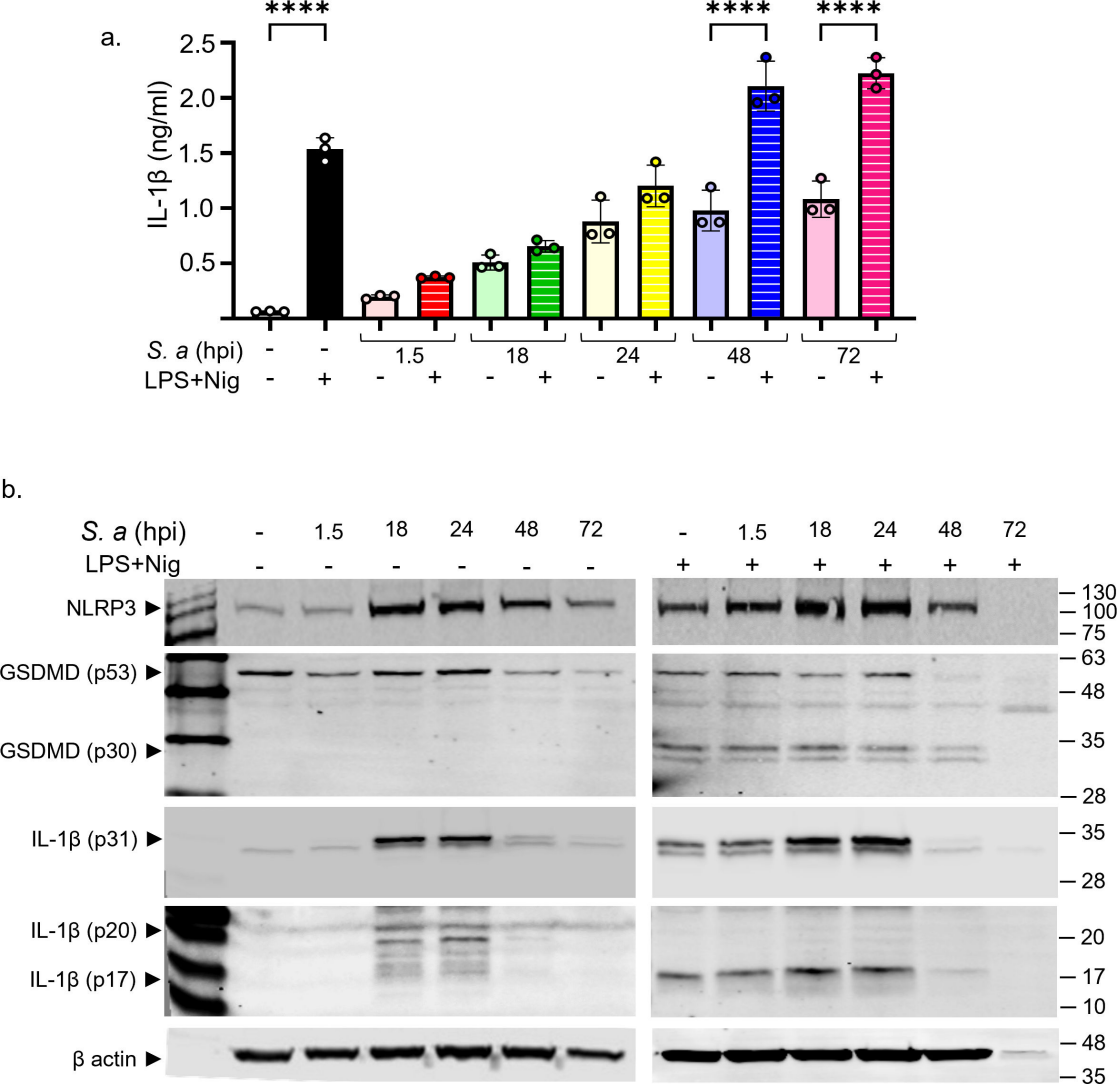
*S. aureus* affects IL-1β and GSDMD maturation. Uninfected and WT BMDMs infected with *S. aureus* were left untreated or primed with LPS for 3 h, then treated with nigericin for 45 min. (**a**) IL-1β. (**b**) Immunoblotting analysis of cell lysates. Data are means ± SD from three biological replicates and are representative of at least three independent experiments. ****P < 0.0001 by one-way ANOVA with Bonferroni’s *post hoc* test.

The NLRP3 inflammasome promotes the uptake of the DNA-binding dye Sytox Green (32). Accordingly, the kinetics of Sytox Green uptake and, to a lesser extent, IL-1β secretion induced by LPS and nigericin closely mirrored the formation of ASC specks (Fig. 3d through f and 4a). Immunoblotting and qPCR analyses indicated that the expression of NLRP3 and pro-IL-1β, but not of GSDMD, was maximally enhanced by *S. aureus* at 24 hpi before declining at later time points (Fig. 4b; Fig. S7a through c). Notably, the levels of *Il1b* and *Pycard* (which encodes ASC) were inversely correlated (Fig. S8a through c). Cleaved GSDMD amino-terminal fragments (p30) and mature IL-1β (p17) were readily detected in samples from LPS and nigericin-treated uninfected and infected BMDMs (Fig. 4b). Unexpectedly, *S. aureus* did not promote GSDMD cleavage and induced IL-1β species of varying molecular weights, ranging from 17 kDa (mature IL-1β) to 31 kDa (pro-IL-1β) (Fig. 4b). Technical issues, including cell loss and overestimation of protein concentrations, could account for the lack of protein detection at the 72 hpi time point. In any case, these findings suggest that *S. aureus* initially suppresses NLRP3 inflammasome activation, delaying ASC speck formation and downstream responses, which are partially restored at later stages of infection.

**Fig 5:**
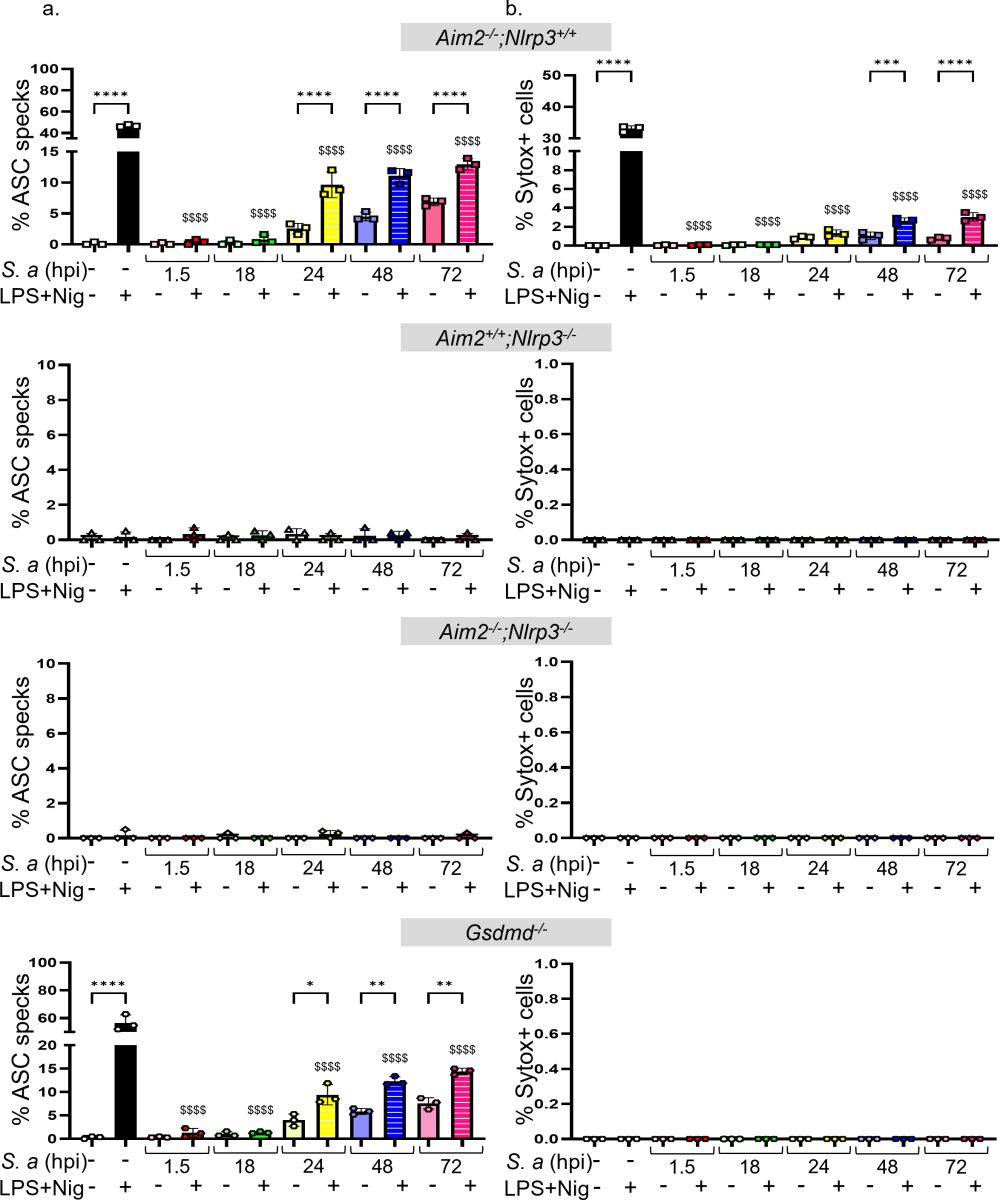
Stimulation with LPS and nigericin induces NLRP3-dependent ASC speck formation and Sytox Green uptake, albeit to a lesser extent, and this response is inhibited by *S. aureus. Aim2^+/+^;Nlrp3^+/+^*, *Aim2^+/+^;Nlrp3^-/-^, Aim2^-/-^;Nlrp3^+/+^, Aim2^-/-^;Nlrp3^-/-^,* and *Gsdmd^-/-^* BMDMs were left uninfected or infected with *S. aureus* for up to 72 h. (**a**) ASC specks. (**b**) Sytox Green. Data are means ± SD from three biological replicates and are representative of at least three independent experiments. **P < 0.01; ***P < 0.001; ****P < 0.0001 by one-way ANOVA with Bonferroni’s *post hoc* test. Comparison of LPS + Nig positive control with infection samples treated with LPS + Nig shown as $$$$ *P* < 0.0001 by one-way ANOVA with Bonferroni’s post hoc test.

To further demonstrate that *S. aureus* primarily modulates the inflammasome assembled by NLRP3, but not AIM2, we examined the activation of these inflammasomes in response to LPS and nigericin in both uninfected and infected BMDMs, assessing each sensor individually and in combination. We also analyzed the impact of GSDMD deficiency on *S. aureus*-induced outcomes. We found that the formation of ASC specks induced by LPS and nigericin was inhibited by *S. aureus* in the presence of NLRP3 but completely abolished in its absence, regardless of AIM2 or GSDMD (Fig. 5a; Fig. S9a and b). Accordingly, the uptake of Sytox Green, although marginal, was observed only in *Aim2^-/-^;Nlrp3^+/+^* BMDMs, but not in *Aim2^+/+^;Nlrp3^-/-^*, *Aim2^-/-^;Nlrp3^-/-^*, or *Gsdmd^-/-^* BMDMs (Fig. 5b; Fig. S10a and b). These results confirm that NLRP3 is responsible for sensing *S. aureus* during infection, whereas AIM2 contributes minimally to this response under these experimental settings.

### Activation of the NLRP3 inflammasome is not restricted to BMDMs containing *S*. *aureus* during late stages of infection

**Fig 6:**
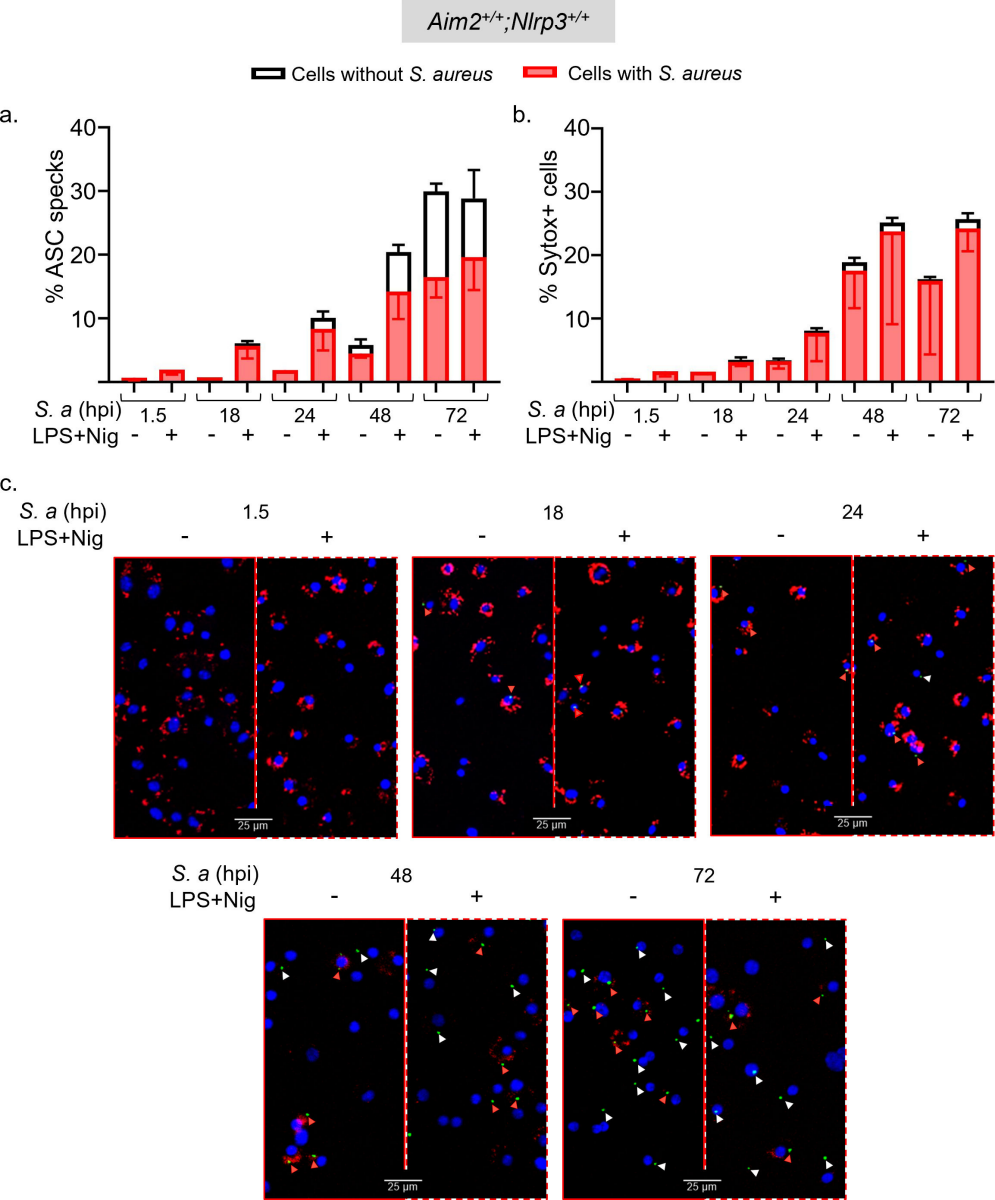
The NLRP3 inflammasome is suppressed during the early stages of *S. aureus* infection, but its activation is not restricted to BMDMs with internalized *S. aureus* during late stages of infection. BMDMs from *ASC-citrine* mice (to assess ASC speck formation) and WT mice (to measure Sytox Green uptake) were left uninfected or infected with *S. aureus* for up to 72 h and were left untreated or treated sequentially with LPS for 3 h and nigericin for 45 min. (**a**) ASC specks were quantified with respect to their colocalization with the *S. aureus* signal within a BMDM. (**b**) Sytox Green-stained nuclei were quantified with respect to the *S. aureus* signal within a BMDM. (**c**) Representative images showing ASC speck formation. Red arrows denote ASC specks in cells with *S. aureus*, while white arrows denote ASC specks in cells without *S. aureus*. Note that the data in Fig. 3 were reanalyzed here to differentiate ASC^+^ cells harboring detectable *S. aureus* from those without detectable bacteria. Data are means ± SD from experimental triplicates and are representative of at least three independent experiments.

To investigate the mechanisms underlying *S. aureus* regulation of the NLRP3 inflammasome pathway, we examined whether the presence of this bacterium inside the cell is necessary for ASC speck formation or Sytox Green uptake. Although the formation of ASC specks was marginal at the early phases of infection, up to 24 hpi, it was confined to *S. aureus*-engulfed BMDMs (Fig. 6a and c). As the infection progressed, BMDMs without this bacterium also became ASC speck^+^ (Fig. 6a and c). Intriguingly, Sytox Green uptake was predominantly restricted to BMDMs containing *S. aureus* (Fig. 6b). These findings suggest that the internalization of *S. aureus* by BMDMs is essential for the initial activation of the inflammasome; however, as the infection progresses, specks were also visible in cells without detectable bacteria.

### ASC speck formation *in vivo* is not confined to macrophages harboring *S. aureus,* and NLRP3 deficiency does not affect *S. aureus* osteomyelitis

**Fig 7:**
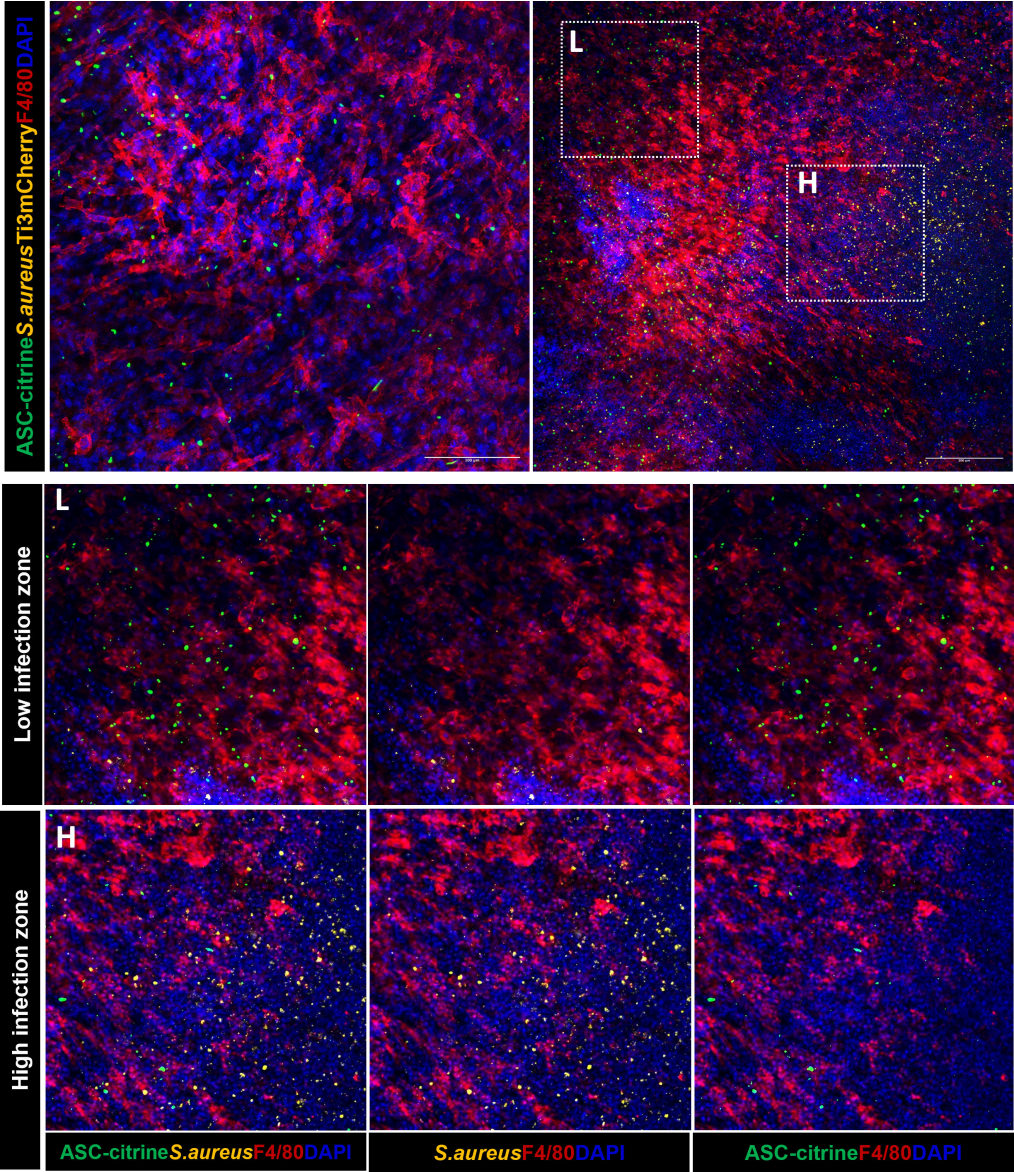
ASC speck formation *in vivo* is not confined to macrophages harboring *S. aureus*. ASC-citrine mice were inoculated with 1 × 10⁶ CFU of mCherry-labeled Ti3 *S. aureus* applied directly to the calvaria. Calvarial samples were incubated for 3 days at 4°C with rabbit anti-F4/80 antibody, followed by overnight incubation at 4°C with Alexa Fluor 647-conjugated anti-rabbit IgG. After extensive washing, nuclei were counterstained with DAPI, and the samples were imaged using a confocal microscope. Squares with dashed lines denote magnified areas shown in the lower panels. L: low infection zone; H: high infection zone.

To investigate the *S. aureus*-inflammasome interaction *in vivo*, we inoculated mCherry-labeled *S. aureus* onto the calvaria of *Asc-citrine* mice and immunostained the tissue for the macrophage marker F4/80. We found that ASC specks were more abundant in regions with lower levels of infection (Fig. 7). Furthermore, ASC specks were visualized in macrophages that did not contain *S. aureus*. Thus, consistent with our *in vitro* observations, ASC speck formation *in vivo* is not confined to macrophages harboring *S. aureus*.

To evaluate whether the NLRP3 inflammasome influences *S. aureus* disease pathogenesis *in vivo*, we used a murine model of osteomyelitis in which injection of bacterium into the femoral diaphysis induces local bone destruction that is partially dependent on IL-1 signaling (33–35). Enumeration of CFUs from bone homogenates revealed no significant differences between *Nlrp3^+/+^* and *Nlrp3^-/-^* mice at day 14 post-infection (Fig. S11a). Micro-computerized tomography (µCT) analysis showed comparable loss of cortical and trabecular bone in *Nlrp3^+/+^* and *Nlrp3^-/-^* mice (Fig. S11b and c). Together, these results indicate that NLRP3 deficiency does not affect the pathogenesis of *S. aureus*-induced osteomyelitis.

## DISCUSSION

Previous studies indicate that NLRP3 and ASC are required for macrophages to activate caspase-1 and secrete IL-1β in response to signals induced by *S. aureus* (14). Subsequent studies revealed that the NLRP3 inflammasome senses *S. aureus* peptidoglycan, hemolysins, and lipoproteins (13, 30, 36). In addition, caspase-1 was shown to promote phagosome-mediated killing of *S. aureus* through the acidification of these organelles (37). In contrast, other studies suggest that *S. aureus* virulence factors, such as alpha toxin, peptidoglycan O-acetyltransferase A, and protein A, limit pathogen recognition and intracellular pattern exposure and exploit both caspase-1 and caspase-11 to evade mitochondrial ROS-mediated killing (38–41). Thus, conflicting findings have been reported regarding the role that inflammasomes play in determining the fate of *S. aureus* in macrophages. These discrepancies may arise from differences in the *S. aureus* strains used and from variations in experimental conditions, including multiplicity of infection, infection duration, and the timing of immune responses. Delayed inflammasome activation may result from the expression or accumulation of pore-forming toxins (e.g., α-toxin and phenol-soluble modulins) and bacterial cytolysins, which disrupt host membranes and facilitate inflammasome activation (42, 43). Consistent with this view, *Trueperella pyogenes* has been reported to release membrane vesicles that activate NLRP3 inflammasome signaling pathways in bovine endometrial epithelial cells in a time- and dose-dependent manner (44). In our time-course studies using a clinical *S. aureus* isolate, we found that the NLRP3 inflammasome eventually partially overcomes the evasive strategies by which *S. aureus* suppresses this pathway during the early stages of infection (Fig. S12). Although the specific mechanisms underlying the time-dependent NLRP3 inflammasome activation remain unclear, our data suggest that inhibition of ASC during the early stages of infection represents a plausible step targeted by *S. aureus* virulence factors such as hla and crtN. Ultimately, the absence of robust NLRP3 inflammasome activation in *S. aureus*-infected macrophages is consistent with our *in vivo* data showing that NLRP3 deficiency does not significantly alter bacterial burden control or bone damage during *S. aureus* osteomyelitis.

We observed that ASC specks were initially detected only in BMDMs that harbored *S. aureus* during the early stages of infection, but they were subsequently observed in cells with or without detectable *S. aureus*. Consistently, ASC specks were also identified *in vivo* within macrophages lacking detectable *S. aureus*. These pathogen-free cells may represent bystander macrophages that were never infected, cells that successfully cleared their intracellular bacterial burden, or cells that responded to secreted PAMPs or DAMPs released from neighboring infected cells. In addition, the possibility that ASC specks were released from inflammasome-activated cells and subsequently internalized by bystander cells cannot be excluded, as reported previously (45, 46). Collectively, our findings demonstrate that *S. aureus* exhibits varying degrees of NLRP3 inflammasome inhibition during early and late stages of infection, underscoring the dynamic and complex nature of its interactions with host cells.

The NLRP3 inflammasome was progressively activated during *S. aureus* infection of BMDMs, as indicated by ASC speck formation, caspase-1 activation, and the production of IL-1β species of distinct molecular weights. Although we did not perform mass spectrometry to definitively identify these species, the presence of multiple higher molecular weight IL-1β forms in *S. aureus*-infected BMDMs support the notion that this bacterium can modify host effector molecules. Such intermediate IL-1β species have been previously reported and are thought to result from incomplete cleavage or alternative proteolytic processing, potentially mediated by microbial or host-derived proteases (47). Unlike IL-1β, we did not detect the cleavage of GSDMD into its active p30 fragment. It is possible that full-length GSDMD forms pores in the presence of *S. aureus*, as recently demonstrated in an experimental model unrelated to infection (48). Alternatively, GSDMD may undergo post-translational modifications by the *S. aureus* strain used in this study. For instance, *Shigella flexneri* IpaH7.8 has been reported to ubiquitinate GSDMD in human epithelial cells, thereby preventing its cleavage by caspase-1 and preserving a niche for bacterial survival and growth (49). Additionally, inflammasomes can promote GSDMD-independent secondary pyroptosis and IL-1 release, and GSDMD inhibition can occur independently of canonical pyroptosis (50, 51). In contrast, other studies suggest that GSDMD-mediated pyroptosis contributes to the pathogenesis of *S. aureus*-induced osteomyelitis (52), highlighting the context-dependent role of GSDMD in *S. aureus* infection.

Our study is not without limitations. First, although this work was performed using murine models, inflammasome signaling can differ substantially between murine and human macrophages, both in kinetics and in regulatory checkpoints. Second, while our results suggest that AIM2 does not play a major role during *S. aureus* infection, we did not examine the contribution of other DNA sensors, such as cyclic GMP-AMP synthase (cGAS), or their downstream effectors, including type I interferons. Notably, *Francisella tularensis* induces GSDMD cleavage, and GSDMD deficiency impairs AIM2 inflammasome activation in BMDMs; therefore, it remains possible that AIM2 appears dispensable in our model due to the absence of GSDMD maturation (53). In addition, although multiple studies have shown that numerous *S. aureus* virulence factors activate the NLRP3 inflammasome, this study examined only the effects of hla or crtN deficiency. Despite these caveats, our work provides novel insights into the temporal regulation of NLRP3 inflammasome activity in BMDMs during *S. aureus* infection, revealing a biphasic host response characterized by early suppression followed by delayed, ineffective activation and demonstrating that NLRP3 is dispensable for controlling osteomyelitis.

## MATERIALS AND METHODS

### Study design

This study aimed to determine the response of BMDMs to *S. aureus* infection. To achieve this goal, we used genetically modified mouse strains and various biochemical and imaging approaches.

### Mice

To evaluate the role of inflammasomes in *S. aureus* infection, we purchased WT*,* R26-CAG-ASC-citrine (030744)*, Aim2^-/-^* (013144), and *Nlrp3^-/-^* (021302) mice from The Jackson Laboratory (Sacramento, CA, USA). *Aim2^-/-^* and *Nlrp3^-/-^* mice were intercrossed to generate *Aim2^-/-^;Nlrp3^-/-^* mice. *Gsdmd* knockout (*Gsdmd*^−/−^) mice were kindly provided by Dr. V. M. Dixit (Genentech, South San Francisco, CA, United States of America). All mice were on the C57BL/6J background, and mouse genotyping was performed by PCR as standardized before (54, 55). The Institutional Animal Care and Use Committees of Washington University School of Medicine in St. Louis and Vanderbilt University Medical Center approved all procedures.

### Bacterial strains and growth conditions

All *in vitro* infection experiments were conducted with derivatives of *S. aureus* USA300 clinical isolate Ti3 (56), unless stated. Bacteria were grown in Trypticase soy broth (TSB) overnight at 37°C with shaking at 225 rpm, subcultured at a dilution of 1:100, grown to mid-exponential phase (optical density at 600 nm [OD_600_] of 1.0), and centrifuged at 3,000 rpm for 10 min. The pellets were washed and resuspended in PBS to the desired concentration. To prepare inocula for the *in vivo* osteomyelitis model, WT *S. aureus* strain AH1263 was grown in TSB overnight at 37°C with shaking at 180 rpm, subcultured at a dilution of 1:100, grown to mid-exponential phase, and then resuspended in phosphate-buffered saline (PBS) at a concentration of approximately 5 × 10^8^ CFU/mL.

### Cell cultures

BMDMs were obtained by culturing murine bone marrow cells in α-MEM culture media containing a 10% (vol/vol) conditioned medium from the fibroblastic cell line CMG 14-12 as a source of macrophage colony-stimulating factor (M-CSF) (57), for 4–5 days in a Petri dish as previously described (58). Briefly, nonadherent cells were removed by PBS washes, and adherent BMDMs were detached with trypsin-EDTA and cultured in 10% CMG α-MEM culture media for various experiments. For all experiments, BMDMs were plated at 10^4^ cells per well on a 96-well plate or 10^6^ cells per well on a six-well plate.

### Serum optimization assay

WT BMDMs were cultured in media containing 0%, 1%, or 10% FBS and infected for 30 min at an MOI of 100 at 37°C in 5% CO_2_, washed twice in PBS, and cultured in the same FBS-supplemented media containing gentamicin (0.3 mg/mL) for 1 h to kill extracellular bacteria after which fresh media (α-MEM + M CSF ± FBS without antibiotics) was added to the cells. Infection was quantified by CFU assay at 1.5 hpi and 18 hpi, along with the assessment of IL-1β secretion and cell death based on the release of lactate dehydrogenase (LDH). WT BMDMs cultured in different FBS concentrations were also subjected to known NLRP3 inflammasome activators, LPS and nigericin, as positive controls (59).

### qPCR analysis

RNA was extracted from BMDMs infected with *S. aureus* and was collected at 2, 6, and 21 hpi using the PureLink RNA Mini Kit (Life Technologies) with on-column DNase I treatment, according to the manufacturer’s instructions. RNA quality and concentration were assessed using a NanoDrop spectrophotometer (Thermo Scientific). Complementary DNA (cDNA) was synthesized from 1 µg of total RNA using the High-Capacity cDNA Reverse Transcription Kit (Applied Biosystems). Gene expression was analyzed by SYBR Green–based quantitative real-time PCR (qRT-PCR) using gene-specific primers. Data were normalized to cyclophilin B, and relative mRNA expression was calculated using the 2^ΔΔCt^ method.

### Western blot analysis

Cell extracts were prepared by lysing cells with radioimmunoprecipitation assay (RIPA) buffer (50 mM tris, 150 mM NaCl, 1 mM EDTA, 0.5% NaDOAc, 0.1% SDS, and 1.0% NP-40) and complete protease inhibitor cocktail. Protein concentrations were determined using a protein estimation kit from Bio-Rad, and equal amounts of proteins were subjected to SDS-polyacrylamide gel electrophoresis gels (12%) as previously described (43). Proteins were transferred onto the PVDF membrane and incubated with antibodies against different protein components of the NLRP3 inflammasome pathway, NLRP3, GSDMD (1:1,000; Abcam, MA), IL-1β, α-tubulin, and β-actin (1:2,000; Santa Cruz Biotechnology, TX), overnight at 4°C, followed by incubation for 1 h with respective secondary goat anti-mouse IRDye 800 (Thermo Fisher Scientific, MA) or goat anti-rabbit Alexa Fluor 680 (Thermo Fisher Scientific, MA). The results were visualized using the Odyssey Infrared Imaging System (LI-COR Biosciences, NE).

Immunoblot signals were quantified using ImageJ/Fiji (NIH). Non-saturated images acquired within the linear range were converted to 8-bit grayscale and inverted. Band intensities were measured using identical regions of interest, with local background subtraction from adjacent areas. Signals were normalized to β-actin. Data are expressed relative to control samples.

### LDH assay and IL-1β ELISA

Lytic cell death was assessed by the release of LDH in conditioned medium using the LDH Cytotoxicity Detection Kit (Takara, CA). Conditioned media were collected, and IL-1β (eBioscience, NY) and IL-18 (R&D Systems, MN) levels were quantified using ELISA kits. For time-course infection analysis, culture supernatants were collected at each designated time point, after which fresh media were added to the cells, and infection was continued until the next time point. Therefore, LDH, IL-1β, and IL-18 levels measured at each time point represent the release relative to the preceding infection interval, rather than cumulative release.

### ASC specks assay

*Aim2^+/+^;Nlrp3^+/+^*, *Aim2^+/+^;Nlrp3^-/-^, Aim2^-/-^;Nlrp3^+/+^, Aim2^-/-^;Nlrp3^-/-^,* and *Gsdmd^-/-^* BMDMs were seeded overnight and infected with *S. aureus* at an MOI of 100 and/or stimulated for inflammasome activation with LPS for 3 h before adding 15 μM nigericin for 45 min as previously described^47^. After the appropriate treatments and infection time points, cell culture supernatants were collected, and the cells were washed with PBS and fixed with 4% paraformaldehyde buffer for 10 min at room temperature, followed by permeabilization and staining for ASC using anti-mouse ASC antibody (EMD Millipore) diluted in buffer containing 0.2% Triton and 1% BSA in PBS. After overnight incubation at 4°C, the cells were stained with a secondary antibody (Alexa Fluor 594; Life Technologies) for 30 min and counterstained with Fluoro-gel II containing 4′,6-diamidino-2-phenylindole (Fluoro-Gel, Fisher Scientific Intl. Inc., PA). The formation of ASC specks, indicating inflammasome assembly, was detected using a Leica inverted microscope with a TCS SPE II confocal module and processed using LAS X software (Leica Microsystems Inc., IL). Quantification of ASC specks was conducted using ImageJ.

To assess ASC speck formation *in vivo*, *Asc-citrine* mice were inoculated with 1 × 10⁶ CFU of mCherry-labeled Ti3 *S. aureus* applied directly onto the calvaria. Three days post-infection, mice were perfused under isoflurane with 15–20 mL of PBS and 10% neutral buffered formalin, and calvariae were harvested. Samples were post-fixed overnight in 10% formalin at 4°C, permeabilized with 0.5% Triton X-100 in PBS for 30 min, and blocked overnight at 4°C in Tris buffer containing 10% donkey serum, 0.3% Triton X-100, and 10% DMSO. Calvarial samples were incubated for 3 days at 4°C with rabbit anti-F4/80 antibody (1:250), followed by incubation with Alexa Fluor 647–conjugated anti-rabbit IgG (1:500) overnight at 4°C. After extensive washing, nuclei were counterstained with DAPI (1 µg/mL) for 10 min. Whole-mount calvarial disks were mounted in Fluoromount on 35-mm glass-bottom dishes and imaged using a Nikon spinning disk confocal microscope.

### Murine model of osteomyelitis

All animal experiments were approved by the Vanderbilt University Institutional Animal Care and Use Committee. WT (Strain #000664) and *Nlrp3^-/-^* (strain #021302) mice were obtained from The Jackson Laboratory and subjected to post-traumatic osteomyelitis as previously described (33–35). Briefly, 7- to 8-week-old mice were anesthetized using isoflurane and provided buprenorphine prior to the procedure for analgesia. The left hindlimb and flank were prepared for surgery, and the diaphysis of the femur was exposed using sterile technique. An approximately 1 mm defect was made in one side of the cortical bone using trephination with a 21-gauge needle. Approximately 1 × 10^6^ CFUs of WT *S. aureus* strain AH1263 (60) in 2 μL PBS were injected into the cortical defect using a micropipette. Muscle fascia and skin were then closed with sutures, and the mice were allowed to recover from anesthesia. 14 days post-infection, mice were euthanized. In the event that a femur was fractured during extraction, the sample was excluded from imaging analyses.

### CFU enumeration

*In vitro*: infection of BMDMs by *S. aureus* was quantified by determining the number of colony-forming units (CFUs). Briefly, BMDMs from WT and different genotypes were seeded at 10^6^/well in six-well plates and cultured as described above. To determine the level of intracellular survival, the cells were infected for 30 min at an MOI of 100 at 37°C in 5% CO_2_, washed twice in PBS, and cultured in α-MEM media containing M-CSF and 0.3 mg/mL gentamicin for 1 h to kill extracellular bacteria. Cells were washed twice in PBS to remove the antibiotic and lysed in sterile, ice-cold ultrapure H_2_O at 1.5 hpi time point. For 18 hpi, culture media were replaced after PBS washes, and the infection was continued to 18 h before hypotonic lysis of the cells as described above. Lysates were 10-fold serially diluted, plated on TSB solidified with 1.5% agar (TSA), incubated overnight at 37°C, and CFUs were enumerated. To ensure that extracellular *S. aureus* was effectively killed in all experiments, supernatants from antibiotic-treated cultures were plated on TSA and inspected for colonies after overnight incubation at 37°C.

*In vivo*: at the desired endpoint, femurs were sterilely dissected from euthanized mice, placed in NAVY bead lysis tubes (Next Advance, Troy, NY) containing 500 μL of sterile 1× PBS, and homogenized at 4°C using a Bullet Blender at the highest setting for 3 intervals of 5 min each. Homogenates were serially diluted in sterile 1× PBS and plated on TSA plates. Plates were incubated overnight at 30°C, and CFUs were counted the next day.

### Micro-computed tomography (μCT) of cortical and trabecular bone in femurs

Infected and contralateral femurs were dissected from mice at day 14 post-infection, fixed in 10% neutral buffered formalin for 48 h, and then moved to 70% EtOH before storage at 4°C. Fixed femurs were scanned using a μCT50 (Scanco Medical, Switzerland) instrument and analyzed with μCT Tomography software (Scanco USA, Inc.). Scans were acquired at 10.0 μm voxel size at 70 kV, 200 μA, and an integration time of 350 ms in a 10.24 mm view to result in 1,088 image slices. To accommodate the analysis of trabecular and cortical bone, a region of interest was selected on each femur to encompass the trabecular bone of the distal epiphysis, as well as the entire diaphysis so that the cortical defect into which bacteria were inoculated could be visualized. The proximal epiphysis was excluded. Cortical bone destruction of the infected femur and relative trabecular bone volume of the infected and contralateral femurs were determined by contouring the indicated regions of interest as previously described (33). Briefly, to analyze cortical bone loss and callus formation, 818 slices were contoured, centered around the midpoint of the defect. For trabecular bone analysis, measurements of the distal trabecular bone were standardized to begin 30 slices above the growth plate. A total of 101 slices were analyzed.

### Sytox Green uptake assay

BMDMs were seeded in tissue culture plates and either primed using LPS or given *S. aureus* infection for the indicated time points. The cells were incubated with Hoechst 33342 (1 µg/mL) for 15 min, the media were changed, and treated with nigericin for 45 min. Following stimulation, Sytox Green (Invitrogen) diluted in PBS was added to the wells to reach a final concentration of 10 nM. The cells were incubated for 10–15 min to allow the dye to penetrate the cells. Fluorescence was measured in live cells using a Leica inverted microscope with a TCS SPE II confocal module and processed using LAS X software (Leica Microsystems Inc., IL). The percentage of Sytox Green-positive cells was analyzed and quantified using ImageJ.

### Flow cytometry

BMDMs were seeded in six-well tissue culture plates and challenged with GFP-expressing Ti3-*S. aureus* at an MOI of 1:100 for 30 min. Extracellular bacteria were killed by the addition of gentamicin to the cultures for 1 h. Cells were washed twice in PBS to remove the antibiotic; then, the media were replenished and harvested at the indicated time points. Cells were detached with trypsin-EDTA, washed twice in PBS to remove non-adherent bacteria as previously described (61), and analyzed by flow cytometry (percent fluorescein isothiocyanate [FITC]-positive cells and mean fluorescence intensity of FITC-positive population). Flow cytometry acquisition was performed using the Attune Flow Cytometer system, followed by analysis with FlowJo software (Tree Star, Ashland, Oregon).

### FLICA assay

Active caspase-1 was labeled using the FAM-FLICA Caspase-1 (YVAD) Assay Kit (Immunochemistry Technologies, MN). BMDMs were seeded as described above and stimulated with *S. aureus* in the absence or presence of LPS and nigericin and then treated with 10 μL of 30× FLICA solution. The cells were stained with Hoechst 33342 stain (0.5% wt/vol) and viewed using a Leica inverted microscope with excitation 490 nm and emission > 520 nm for Green fluorescence and excitation 365 nm and emission 480 nm for the Hoechst dye. The percentage of cells staining Green (indicating active caspase-1) was quantified using ImageJ software.

### Statistical analysis

Statistical analysis was performed using the Student’s *t*-test or one-way analysis of variance (ANOVA) with Bonferroni’s multiple comparisons test using GraphPad Prism software. Values are expressed as means ± SEM or as means ± SD, as indicated. *P < 0.05 was considered statistically significant.

## Data Availability

Requests for further information and resources should be directed to Gabriel Mbalaviele (gmbalaviele@wustl.edu).

## SUPPLEMENTAL MATERIAL

Supplemental figures iai.00200-26-s0001.pdf Fig. S1 to S12.

Supplemental material iai.00200-26-s0002.pdf Supplemental figure legends.

ASM does not own the copyrights to Supplemental Material that may be linked to, or accessed through, an article. The authors have granted ASM a non-exclusive, world-wide license to publish the Supplemental Material files. Please contact the corresponding author directly for reuse.
