# Supplementary material for: Staphylococcus aureus inhibits the NLRP3 inflammasome in macrophages to varying degrees during early and late stages of infection
Source: Infect Immun. 2026 Jun 10;94(7):e00200-26. doi: 10.1128/iai.00200-26 (PMC13367054; doi:10.1128/iai.00200-26)
Supplement: Supplemental figures — Fig. S1 to S12. [file iai.00200-26-s0001.pdf]

**Supplementary Fig 1: *S. aureus* is more efficiently killed by BMDMs cultured in media containing a high concentration of FBS.** WT BMDMs were infected with *S. aureus* (*S. a*) under varying serum conditions, 0% FBS (serum starved), 1% FBS (reduced serum) and 10% FBS (normal serum) and analyzed post-infection. a. CFUs from lysates of infected BMDMs were quantified at 1.5 hpi, immediately after gentamicin exposure, or after an additional 16.5 h in culture media (18 hpi). b. LDH release in the conditioned media was measured using the LDH kit. c. IL-1β secretion in the conditioned media were evaluated by ELISA. d. Uninfected BMDMs treated with 1% or 10% FBS were primed with LPS for 3 h followed by 45 min of nigericin treatment. Data are means ± SD from experimental triplicates and are representative of three independent experiments. *p<0.05; **p<0.01; ***p<0.001 by two-way ANOVA (a), one-way ANOVA (b, c) with Bonferroni's *post hoc* test or Student's *t* test (d).

**Supplementary Fig 2: IL-1β secretion in BMDMs is regulated by *S. aureus* in a strain-specific manner.** WT BMDMs were infected with Ti3, heat-killed Ti3, LAC, Δ*hla* LAC, JE2, or Δ*crtN* JE2 *S. aureus* at 100 MOI for 1.5 h, 18 h, or 24 h. The supernatants were analyzed for IL-1β secretion. Data are means ± SD from three biological replicates and are representative of three independent experiments. **p<0.01; ***p<0.001; ****p<0.0001 by one-way ANOVA with Bonferroni's *post hoc* test.

**Supplementary Fig 3: Flow cytometric analysis of *S. aureus*-infected BMDMs.** *Aim2*$^{+/+}$*;Nlrp3*$^{+/+}$, *Aim2*$^{+/+}$*;Nlrp3*$^{-/-}$, *Aim2*$^{-/-}$*;Nlrp3*$^{+/+}$, and *Aim2*$^{-/-}$*;Nlrp3*$^{-/-}$ BMDMs were infected with GFP-tagged *S. aureus.* a. CFU enumeration. b. flow cytometry analysis. c. quantitative tabulation of the frequencies of GFP$^{+}$ cells with respect to total live cells. a. Data are means ± SD from three biological replicates and represents combined results from three independent experiments b. Data are means ± SD from experimental triplicates and are representative of at least three independent experiments.

**Supplementary Fig 4: Effects of *S. aureus* on IL-1β and IL-18 secretion in BMDMs.** WT BMDMs were left uninfected (control) or infected with *S. aureus* for up to 96 h. Uninfected cells sequentially treated with LPS for 3 h and nigericin for 45 min were used as positive controls. The supernatants were analyzed for IL-1β secretion and LDH release. a, b. IL-1β secretion. c. IL-18 secretion. Data are means ± SD from three biological replicates and are representative of three independent experiments. **p<0.01; ***p<0.001; ****p<0.0001 by one-way ANOVA with Bonferroni's *post hoc* test.

**Supplementary Fig 5: ASC specks are not detected in uninfected BMDMs.** BMDMs from *ASC-citrine* mice were maintained in cultures for up to 72 h. The cells were incubated with DAPI (1 µg/ml) for 10 min. ASC specks were visualized under fluorescence microscope. Data images are representative of three independent experiments. Scale bars: 50 µm.

**Supplementary Fig 6: *S. aureus* suppresses the activation of caspase-1 during the early phases of infection of BMDMs, but to a lesser extent during the late phases.** BMDMs were infected with *S. aureus* for up to 48 h. Cells left untreated or treated sequentially with LPS for 3 h and nigericin for 45 min then incubated with 10 µl of 30X FLICA solution followed by the staining with Hoechst 33342. a. FLICA b. Data are means ± SD from experimental triplicates and are representative of at least three independent experiments.

**Supplementary Fig 7: *S. aureus* affects IL-1β and GSDMD maturation.** Uninfected and WT BMDMs infected with *S. aureus* were left untreated or primed with LPS for 3 h, then treated with nigericin for 45 min. Cell lysates were analyzed by immunoblotting. a. NLRP3. c. GSDMD and GSDMD-NT. c. IL-1β. Immunoblot signals were quantified using ImageJ/Fiji (NIH). Signals were normalized to β-actin. Data expressed relative to control samples are means ± SD and are

representative of three independent experiments. *p<0.05; **p<0.01; ***p<0.001; ****p<0.0001 by one-way ANOVA with Bonferroni's *post hoc* test.

**Supplementary Fig 8: *S. aureus* regulates the expression of *Nlrp3*, *Il1b*, and *Pycard* in a time-dependent manner.** RNA was extracted from BMDMs infected with *S. aureus* and were collected at 2, 6, and 21 hpi. Gene expression was analyzed by qPCR. a. *Nlrp3*. *b*. Il1b. c. *Pycard*. Data were normalized to cyclophilin B, and relative mRNA expression was calculated using the $2^{\Delta\Delta Ct}$ method. Data are means ± SD from experimental triplicates and representative of three independent experiments.

**Supplementary Fig 9: The formation of ASC specks induced by LPS and nigericin depends on NLRP3.** *Aim2$^{+/+}$;Nlrp3$^{-/-}$*, *Aim2$^{-/-}$;Nlrp3$^{+/+}$*, *Aim2$^{-/-}$;Nlrp3$^{-/-}$*, and *Gsdmd$^{-/-}$* BMDMs were left uninfected or infected with *S. aureus* for up to 72 h. Cells were left untreated or treated sequentially with LPS for 3 h and nigericin for 45 min. ASC specks were visualized under fluorescence microscope. Data images are representative of three independent experiments.

**Supplementary Fig 10: The uptake of Sytox green induced by LPS and nigericin depends on NLRP3.** *Aim2$^{+/+}$;Nlrp3$^{-/-}$*, *Aim2$^{-/-}$;Nlrp3$^{+/+}$*, *Aim2$^{-/-}$;Nlrp3$^{-/-}$*, and *Gsdmd$^{-/-}$* BMDMs were left uninfected or infected with *S. aureus* for up to 72 h. Cells were left untreated or treated sequentially with LPS for 3 h and nigericin for 45 min. Sytox green was added to the cultures 10 min prior to imaging by confocal microscope. Data images are representative of three independent experiments.

**Supplementary Fig 11: NLRP3-deficiency does not significantly alter bacterial burden control or bone homeostasis during *S. aureus* osteomyelitis.** WT and *Nlrp3$^{-/-}$* mice were subjected to osteomyelitis by intraosseous injection of $10^6$ CFU of *S. aureus.* Infected femurs

were extracted on day 14 post-infection. a. Femurs were homogenized for CFU enumeration. Dotted lines indicate the $Log_{10}$ transformed limits of detection. $Log_{10}$ transformed CFU/femur values were compared between genotypes by multiple Mann-Whitney tests with correction for multiple comparisons. No significant differences were detected. WT: n = 5 mice; *Nlrp3*$^{-/-}$: n = 4 mice (1 sample tube exploded in the blender). Error is plotted as standard deviation. b. Cortical bone loss was calculated using μCT and values were compared between genotypes by unpaired t-test. No significant differences were detected. n = 5 mice per genotype. c. Trabecular bone volume was assessed using μCT to compute trabecular bone volume over total volume (% BV/TV). % BV/TV was compared between genotypes for the contralateral and infected femurs using Mann-Whitney U-test with correction for multiple comparisons. No significant differences were found. WT: n = 5 mice (1 femur fractured and could not be used for BV/TV analysis); *Nlrp3*$^{-/-}$: n = 4 mice.

**Supplementary Fig 12: Graphical abstract.** *S. aureus* initially suppresses NLRP3 inflammasome activation in BMDMs (0-18 hpi). As infection progresses, BMDMs partially overcome this inhibition (24-96 hpi).
