# Supplementary material for: Staphylococcus aureus inhibits the NLRP3 inflammasome in macrophages to varying degrees during early and late stages of infection
Source: Infect Immun. 2026 Jun 10;94(7):e00200-26. doi: 10.1128/iai.00200-26 (PMC13367054; doi:10.1128/iai.00200-26)
Supplement: Supplemental material — Supplemental figure legends. [file iai.00200-26-s0002.pdf]

Fig S1

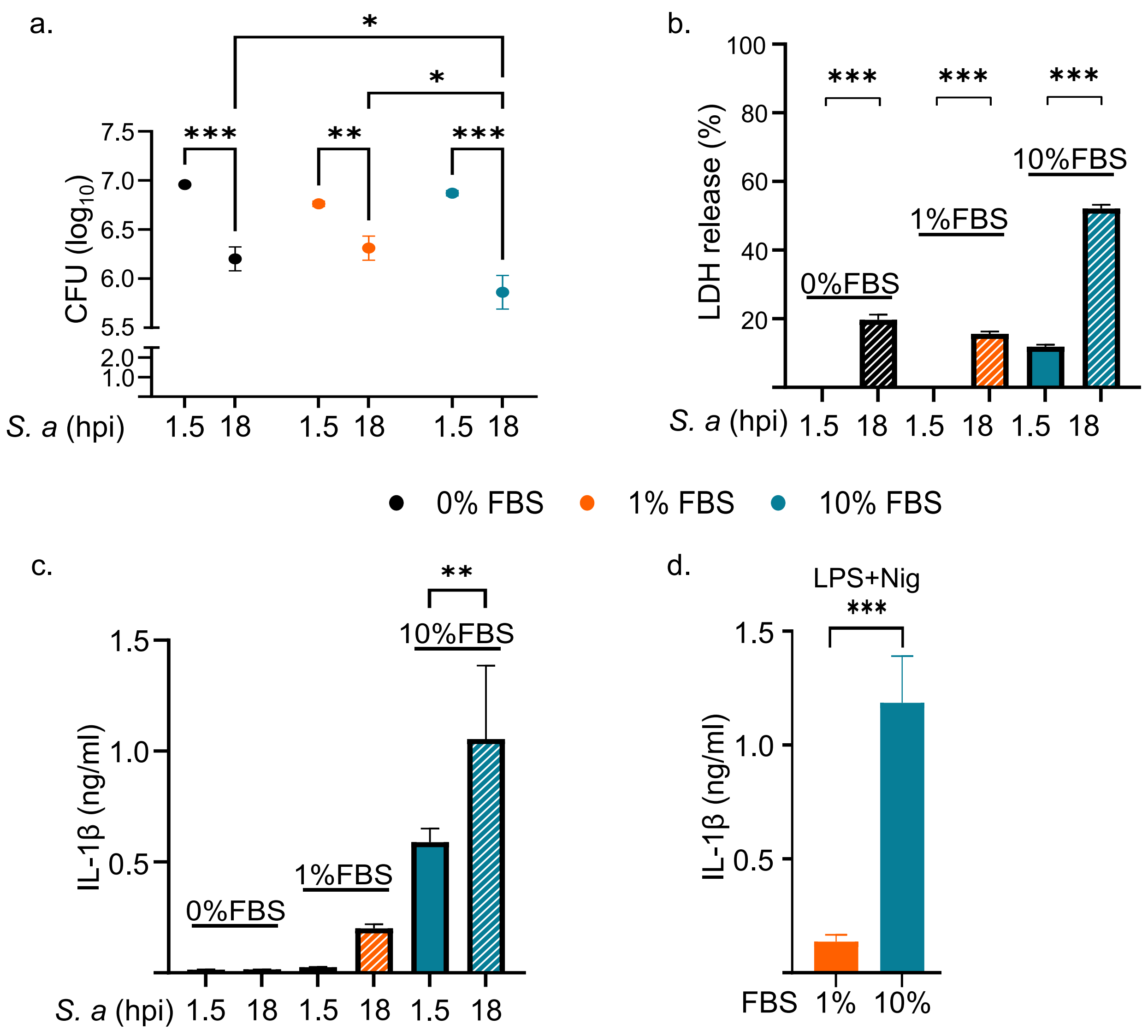

a.
CFU (log10)
7.5
7.0
6.5
6.0
5.5
2.0
1.0
***
**
***
*
*
S. a (hpi) 1.5 18 1.5 18 1.5 18
b.
LDH release (%)
100
80
60
40
20
***
***
***
0%FBS
1%FBS
10%FBS
S. a (hpi) 1.5 18 1.5 18 1.5 18
0% FBS
1% FBS
10% FBS
c.
IL-1β (ng/ml)
1.5
1.0
0.5
**
0%FBS
1%FBS
10%FBS
S. a (hpi) 1.5 18 1.5 18 1.5 18
d.
LPS+Nig
***
IL-1β (ng/ml)
1.5
1.0
0.5
FBS 1% 10%

Suppl 2

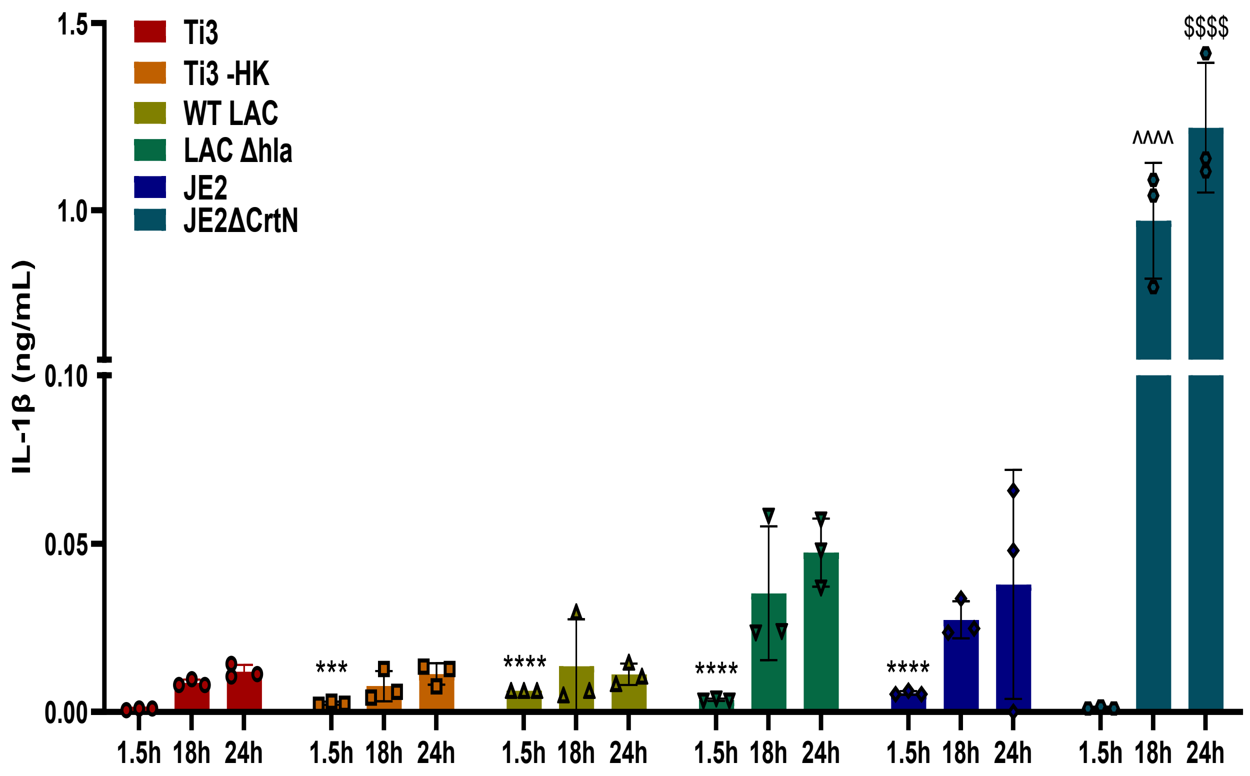

Ti3
Ti3 -HK
WT LAC
LAC Δhla
JE2
JE2ΔCrtN
IL-1β (ng/mL)
1.5
1.0
0.10
0.05
0.00
1.5h 18h 24h 1.5h 18h 24h 1.5h 18h 24h 1.5h 18h 24h 1.5h 18h 24h 1.5h 18h 24h
***
****
****
****
^^^^
$$$$

Suppl 3

a.

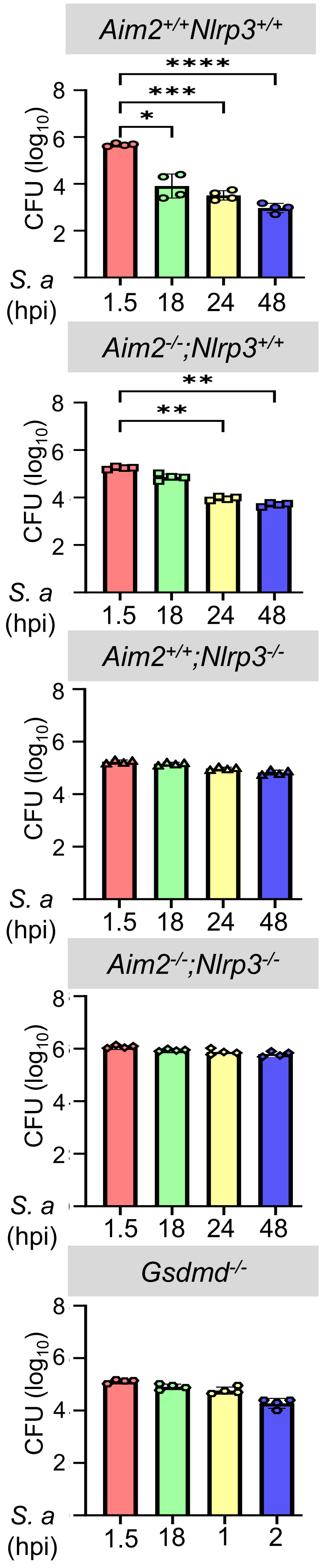

b.

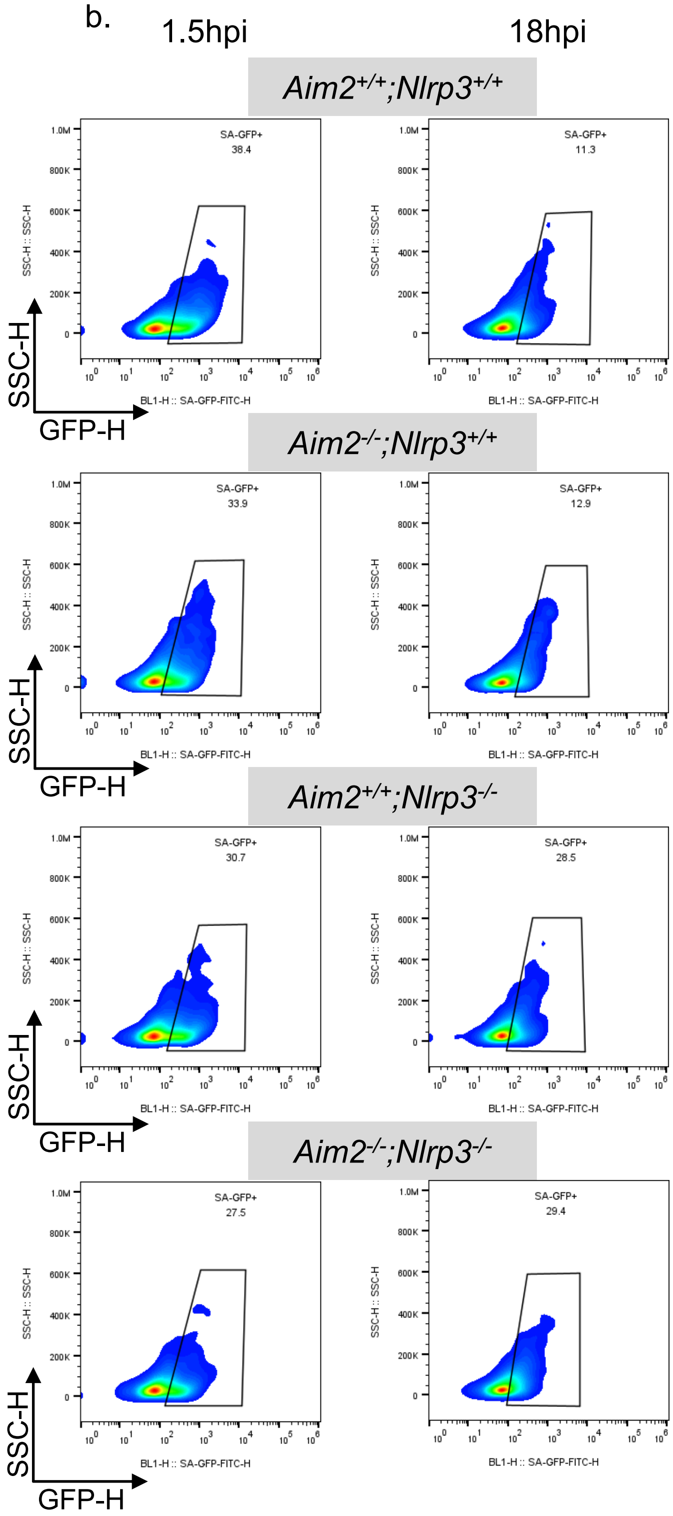

c.

<u>**Genotype**</u>	<u>***S. a* infection**</u>	
	**Freq of GFP+ cells**	
	<u>1.5hpi</u>	<u>18hpi</u>
Aim2+/+;Nlrp3+/+	37.4	13.5
Aim2-/-;Nlrp3+/+	39.9	13.2
Aim2+/+;Nlrp3-/-	30.4	29.2
Aim2-/-;Nlrp3-/-	30.8	28.0

Suppl 4

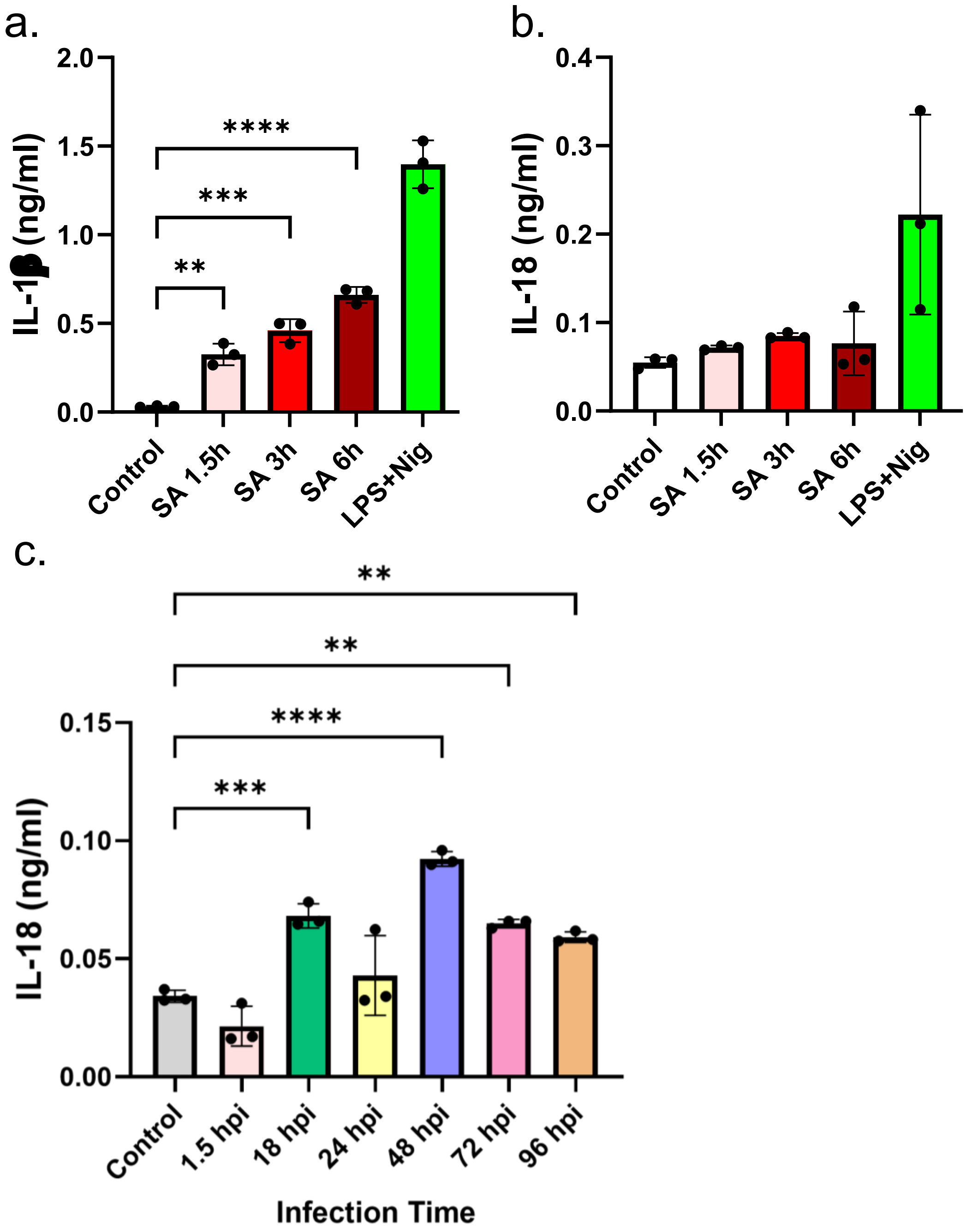

a.
IL-1β (ng/ml)
2.0
1.5
1.0
0.5
0.0
****
***
**
Control
SA 1.5h
SA 3h
SA 6h
LPS+Nig
b.
IL-18 (ng/ml)
0.4
0.3
0.2
0.1
0.0
Control
SA 1.5h
SA 3h
SA 6h
LPS+Nig
c.
**
**
****
***
IL-18 (ng/ml)
0.15
0.10
0.05
0.00
Control
1.5 hpi
18 hpi
24 hpi
48 hpi
72 hpi
96 hpi
Infection Time

Suppl 5

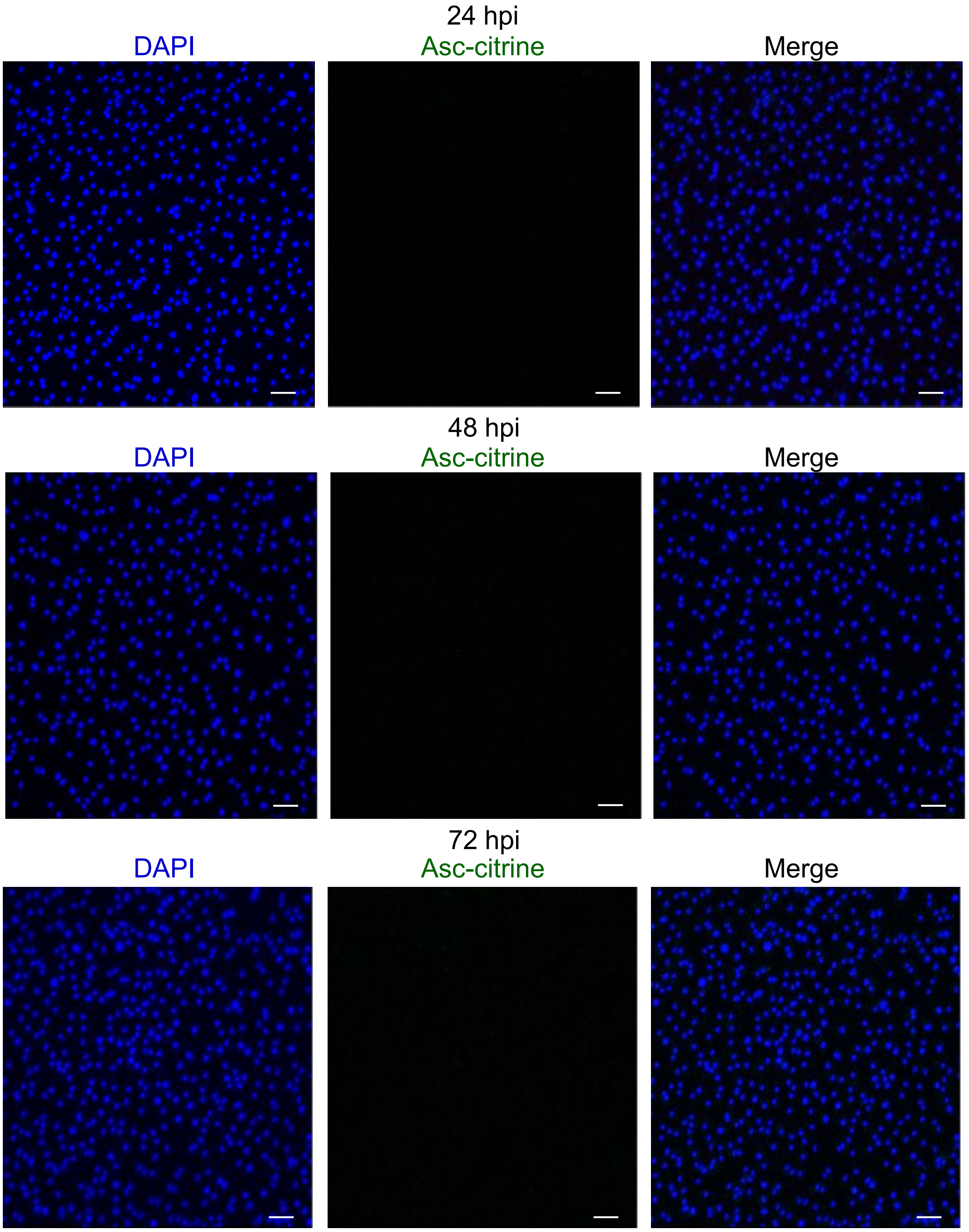
24 hpi
DAPI
Asc-citrine
Merge
48 hpi
DAPI
Asc-citrine
Merge
72 hpi
DAPI
Asc-citrine
Merge

Suppl 6

Aim2$^{+/+}$;*Nlrp3*$^{+/+}$

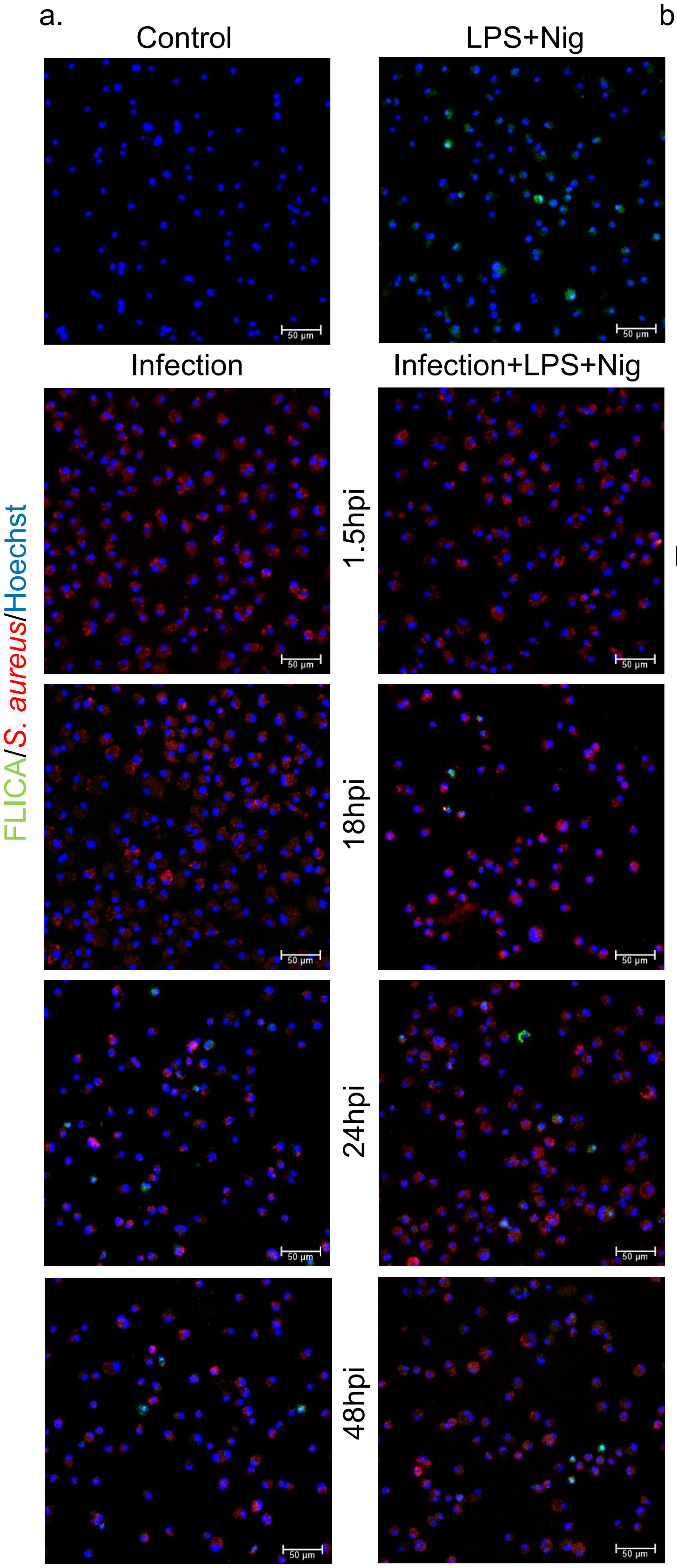

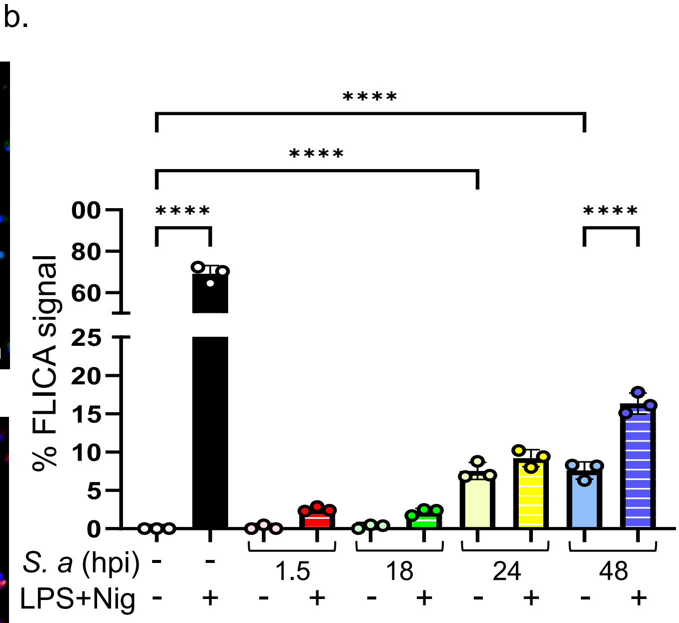

Suppl 7

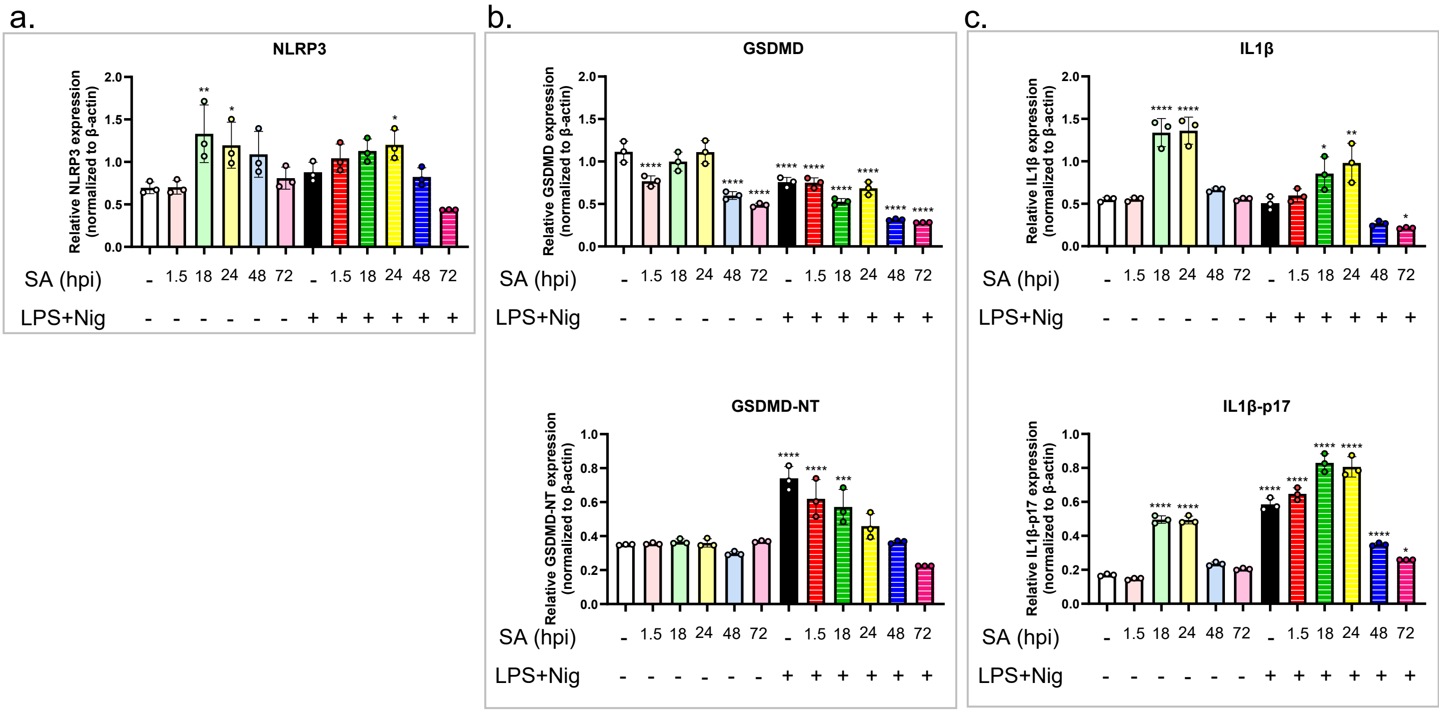

a.
NLRP3
Relative NLRP3 expression (normalized to β-actin)
SA (hpi)
LPS+Nig
b.
GSDMD
Relative GSDMD expression (normalized to β-actin)
GSDMD-NT
Relative GSDMD-NT expression (normalized to β-actin)
c.
IL1β
Relative IL1β expression (normalized to β-actin)
IL1β-p17
Relative IL1β-p17 expression (normalized to β-actin)

Suppl 8

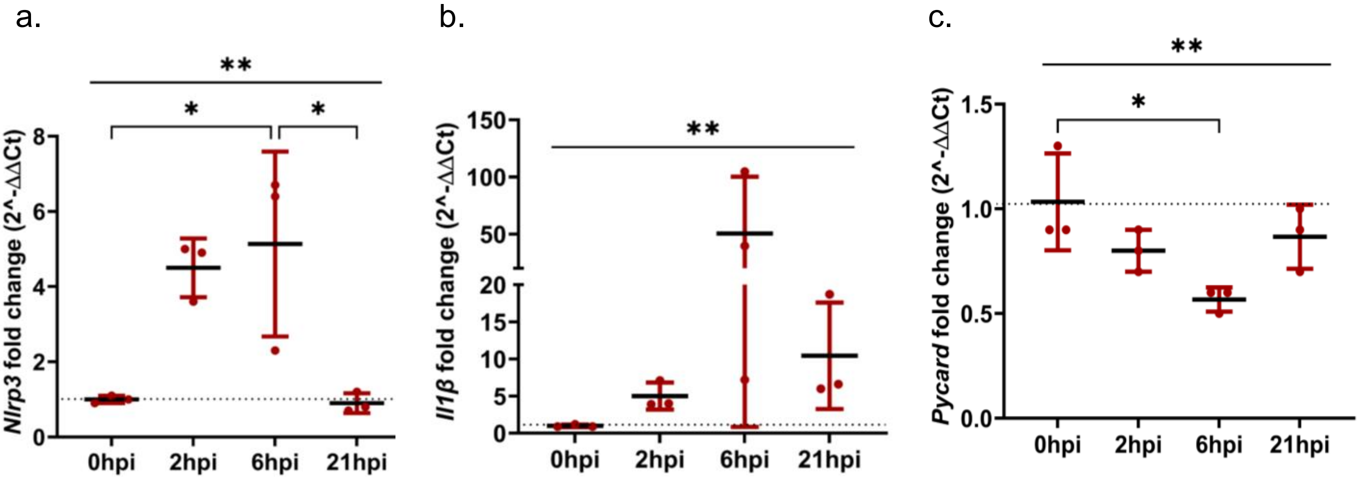
a.
b.
c.
**
*
*
Nlrp3 fold change (2^-ΔΔCt)
0
2
4
6
8
0hpi
2hpi
6hpi
21hpi
Il1β fold change (2^-ΔΔCt)
0
5
10
15
20
50
100
150
Pycard fold change (2^-ΔΔCt)
0.0
0.5
1.0
1.5

Suppl 9

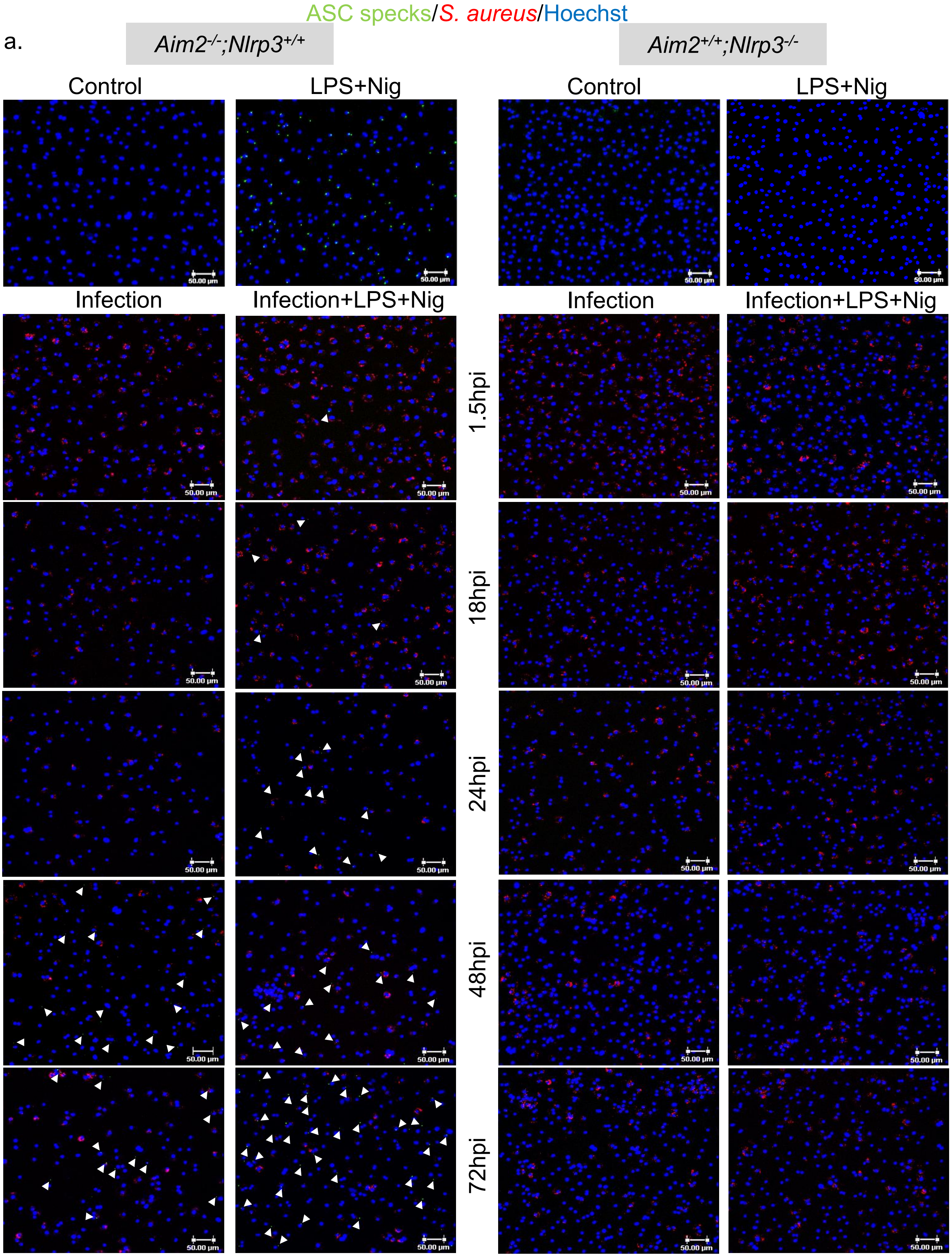

ASC specks/S. aureus/Hoechst
a.
Aim2-/-;Nlrp3+/+
Aim2+/+;Nlrp3-/-
Control
LPS+Nig
Control
LPS+Nig
Infection
Infection+LPS+Nig
Infection
Infection+LPS+Nig
1.5hpi
18hpi
24hpi
48hpi
72hpi
1.5hpi
18hpi
24hpi
48hpi
72hpi
50.00 μm

Suppl 9

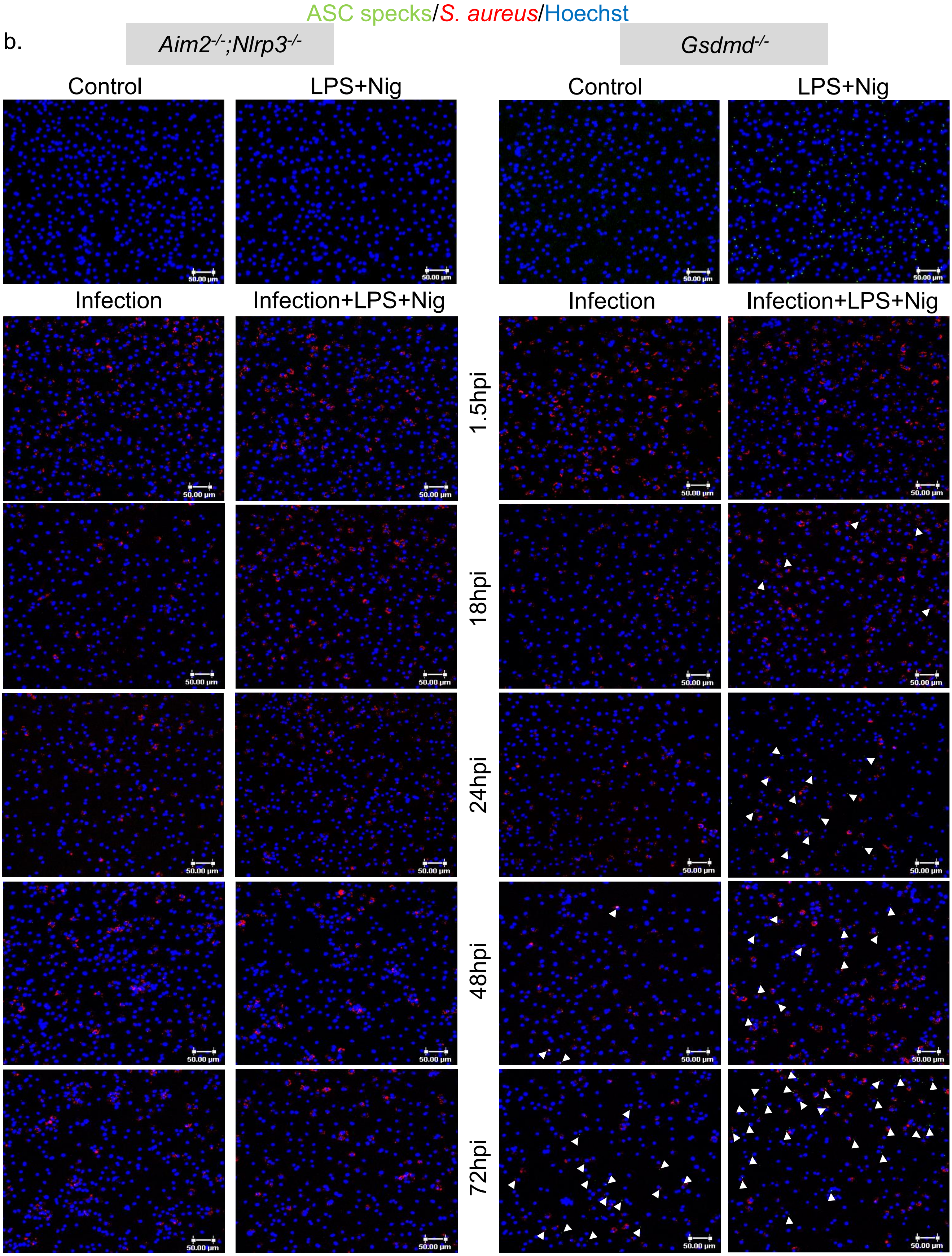

ASC specks/S. aureus/Hoechst
b.
Aim2-/-;Nlrp3-/-
Gsdmd-/-
Control
LPS+Nig
Control
LPS+Nig
Infection
Infection+LPS+Nig
Infection
Infection+LPS+Nig
1.5hpi
18hpi
24hpi
48hpi
72hpi
50.00 µm

Suppl 10

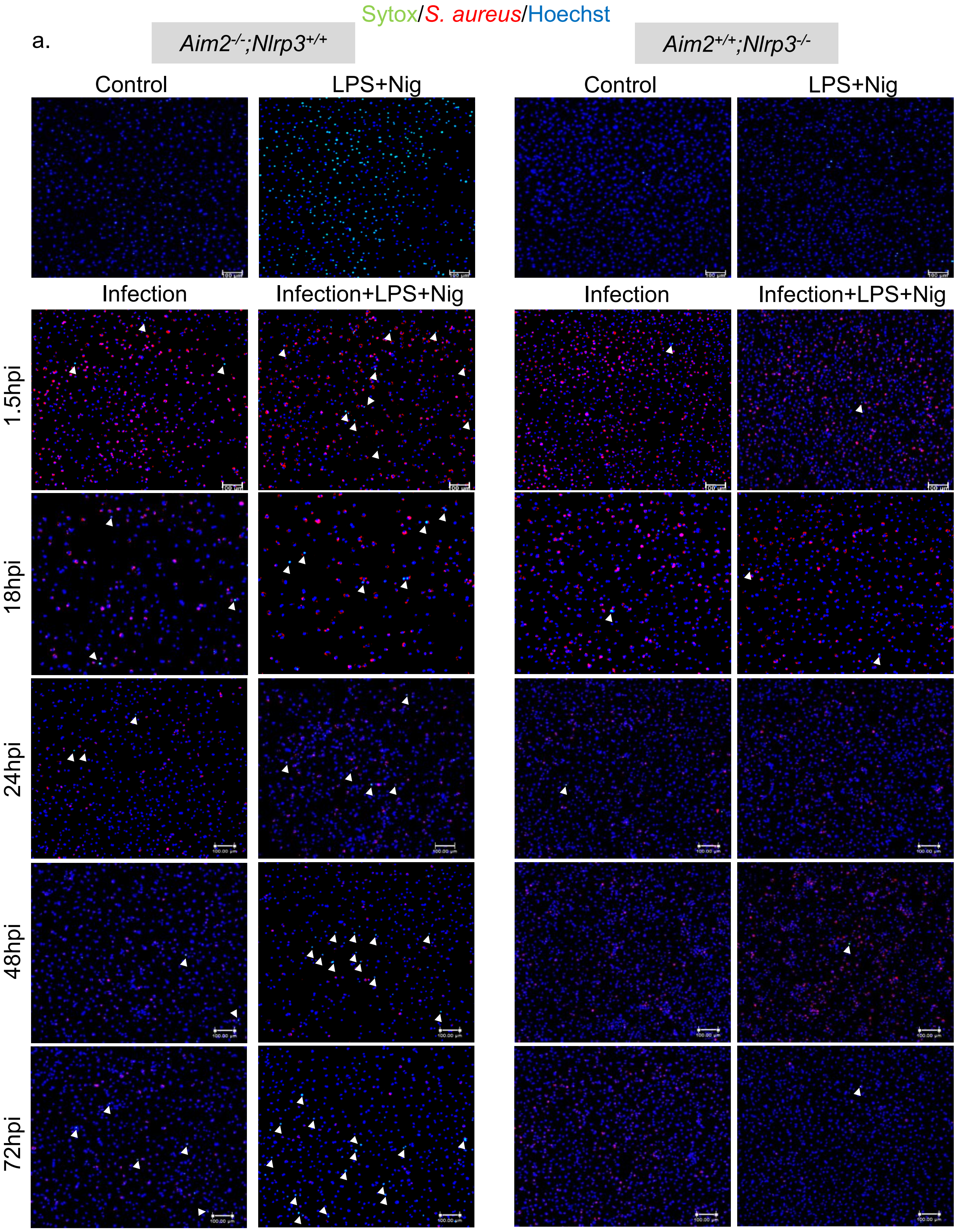

Sytox/S. aureus/Hoechst
a.
Aim2-/-;Nlrp3+/+
Aim2+/+;Nlrp3-/-
Control
LPS+Nig
Control
LPS+Nig
Infection
Infection+LPS+Nig
Infection
Infection+LPS+Nig
1.5hpi
18hpi
24hpi
48hpi
72hpi

Suppl 10

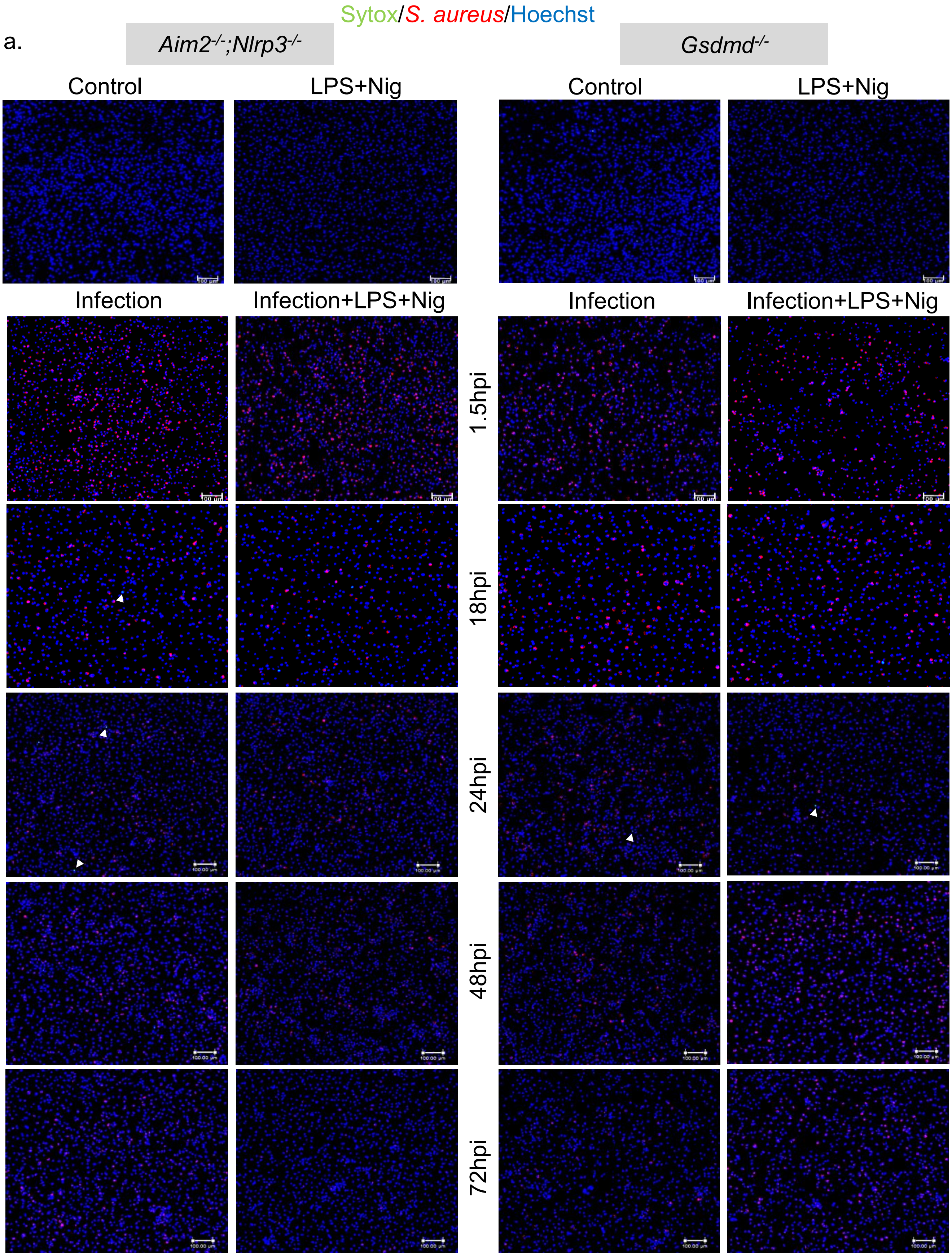
Sytox/S. aureus/Hoechst
a.
Aim2-/-;Nlrp3-/-
Gsdmd-/-
Control
LPS+Nig
Control
LPS+Nig
Infection
Infection+LPS+Nig
Infection
Infection+LPS+Nig
1.5hpi
18hpi
24hpi
48hpi
72hpi

Suppl 11

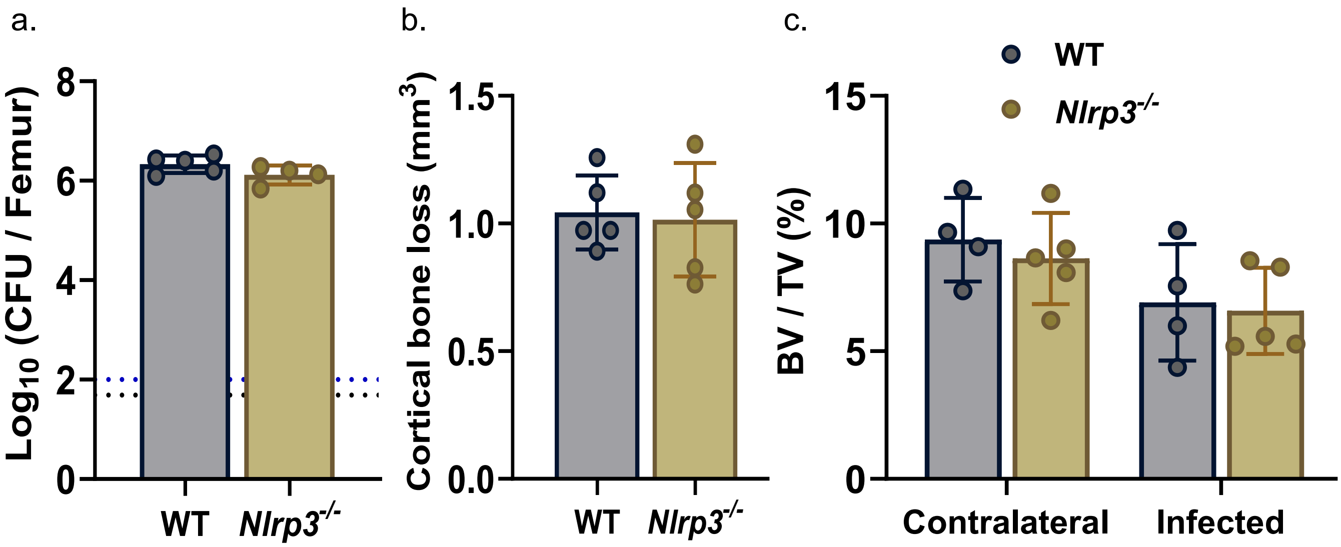
a.
b.
c.
Log10 (CFU / Femur)
8
6
4
2
0
WT
Nlrp3-/-
Cortical bone loss (mm3)
1.5
1.0
0.5
0.0
WT
Nlrp3-/-
WT
Nlrp3-/-
BV / TV (%)
15
10
5
0
Contralateral
Infected

Suppl 12

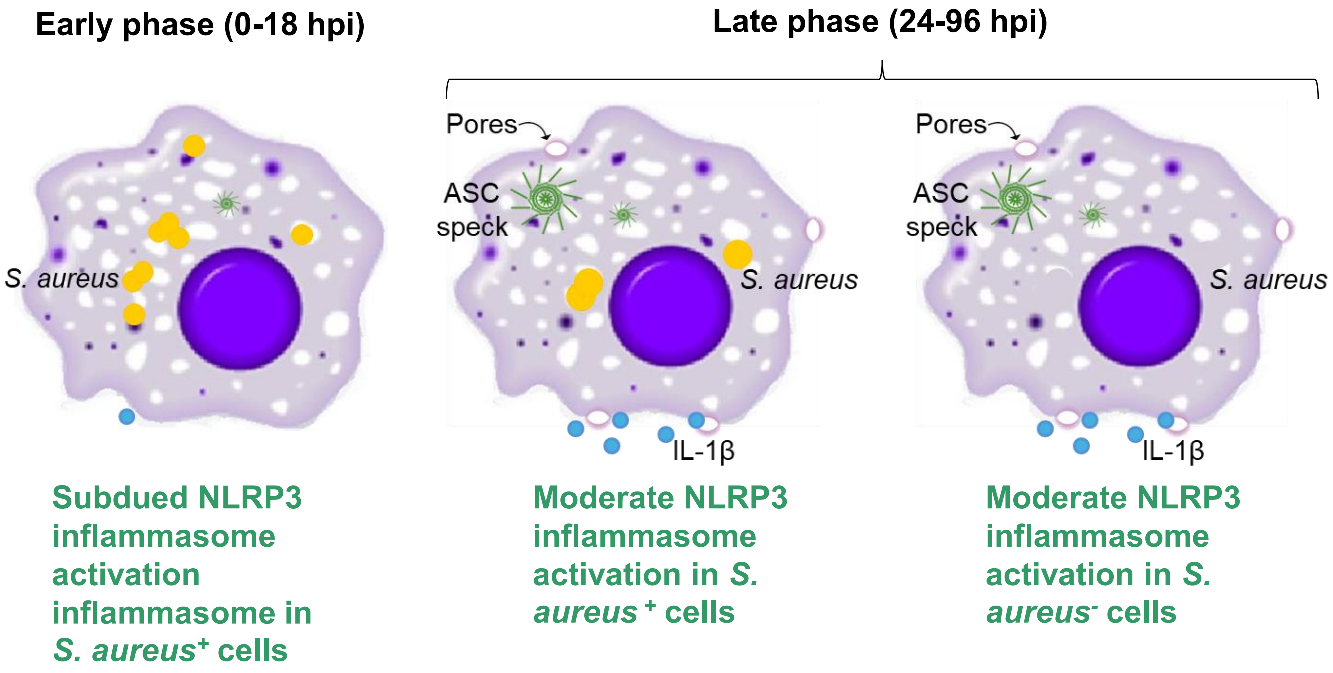

Early phase (0-18 hpi)
Late phase (24-96 hpi)
S. aureus
Pores
ASC speck
S. aureus
IL-1β
Pores
ASC speck
S. aureus
IL-1β
Subdued NLRP3 inflammasome activation inflammasome in S. aureus+ cells
Moderate NLRP3 inflammasome activation in S. aureus + cells
Moderate NLRP3 inflammasome activation in S. aureus- cells
